# On the Occurrence and Multimerization of Two-Polypeptide Phage Endolysins Encoded in Single Genes

**DOI:** 10.1128/spectrum.01037-22

**Published:** 2022-07-25

**Authors:** Daniela Pinto, Raquel Gonçalo, Mariana Louro, Marta Sousa Silva, Guillem Hernandez, Tiago N. Cordeiro, Carlos Cordeiro, Carlos São-José

**Affiliations:** a Research Institute for Medicines (iMed.ULisboa), Faculdade de Farmácia da Universidade de Lisboa, Lisbon, Portugal; b Laboratório de FT-ICR e Espectrometria de Massa Estrutural, MARE – Marine and Environmental Sciences Centre, Faculdade de Ciências da Universidade de Lisboa, Lisbon, Portugal; c Instituto de Tecnologia Química e Biológica António Xavier, Universidade Nova de Lisboa, Oeiras, Portugal; University of Tennessee

**Keywords:** bacteriophage, endolysin, protein isoforms, cell wall, protein complex, bacteriophage lysis, overlapping genes

## Abstract

Bacteriophages (phages) and other viruses are extremely efficient in packing their genetic information, with several described cases of overlapping genes encoded in different open reading frames (ORFs). While less frequently reported, specific cases exist in which two overlapping ORFs are in frame and share the stop codon. Here, we studied the occurrence of this genetic arrangement in endolysins, the phage enzymes that cut the bacterial cell wall peptidoglycan to release the virion progeny. After screening over 3,000 endolysin sequences of phages infecting Gram-positive bacteria, we found evidence that this coding strategy is frequent in endolysin genes. Our bioinformatics predictions were experimentally validated by demonstrating that two polypeptides are indeed produced from these genes. Additionally, we show that in some cases the two polypeptides need to interact and multimerize to generate the active endolysin. By studying in detail one selected example, we uncovered a heteromeric endolysin with a 1:5 subunit stoichiometry that has never been described before. Hence, we conclude that the occurrence of endolysin genes encoding two polypeptide isoforms by in-frame overlapping ORFs, as well as their organization as enzymatic complexes, appears more common than previously thought, therefore challenging the established view of endolysins being mostly formed by single, monomeric polypeptide chains.

**IMPORTANCE** Bacteriophages use endolysins to cleave the host bacteria cell wall, a crucial event underlying cell lysis for virion progeny release. These bacteriolytic enzymes are generally thought to work as single, monomeric polypeptides, but a few examples have been described in which a single gene produces two endolysin isoforms. These are encoded by two in-frame overlapping ORFs, with a shorter ORF being defined by an internal translation start site. This work shows evidence that this endolysin coding strategy is frequent in phages infecting Gram-positive bacteria, and not just an eccentricity of a few phages. In one example studied in detail, we show that the two isoforms are inactive until they assemble to generate a multimeric active endolysin, with a 1:5 subunit stoichiometry never described before. This study challenges the established view of endolysins, with possible implications in their current exploration and design as alternative antibacterials.

## INTRODUCTION

Simpler organisms, like bacteria and viruses, are frequently under selective pressure to reduce their genomes and become more efficient in packing their genetic information (1). In viruses (including phages, viruses that infect bacteria) gene overlap is frequent (1–6), with overlapping genes whose ORFs are in different reading frames being a common arrangement. In this case, genes share part of their sequences, but their protein products do not. Still thought of as an exception is the occurrence of genes with two in-frame overlapping ORFs resulting from the presence of an in-frame, internal translation start site (iTSS). These genes can be thought of as having two alternative start codons, each with its own ribosome binding site (RBS). Such a gene can be expressed in two isoforms sharing part of their amino acid (AA) sequence: one full-length polypeptide encoded by the longest ORF and a shorter polypeptide, initiated at the iTSS, corresponding to a C-terminal part of the full-length product.

We chose to study this phenomenon in phage endolysins, as it has already been identified in phages infecting Mycobacterium, Staphylococcus, *Enterococcus*, *Clostridium* and *Clostridioides* (7–11). Endolysins are enzymes that cut the peptidoglycan mesh that forms the bacterial cell wall. In their natural context, they act at the end of phage infection to destroy the host cell wall, leading to osmotic cell lysis and escape of the phage progeny (12–14). Because they can cause bacteriolysis, endolysins have been gathering significant interest from the scientific community as possible alternative antimicrobials against antibiotic resistant bacteria, namely, because of their low probability to trigger resistance development (15–17).

**FIG 1:**
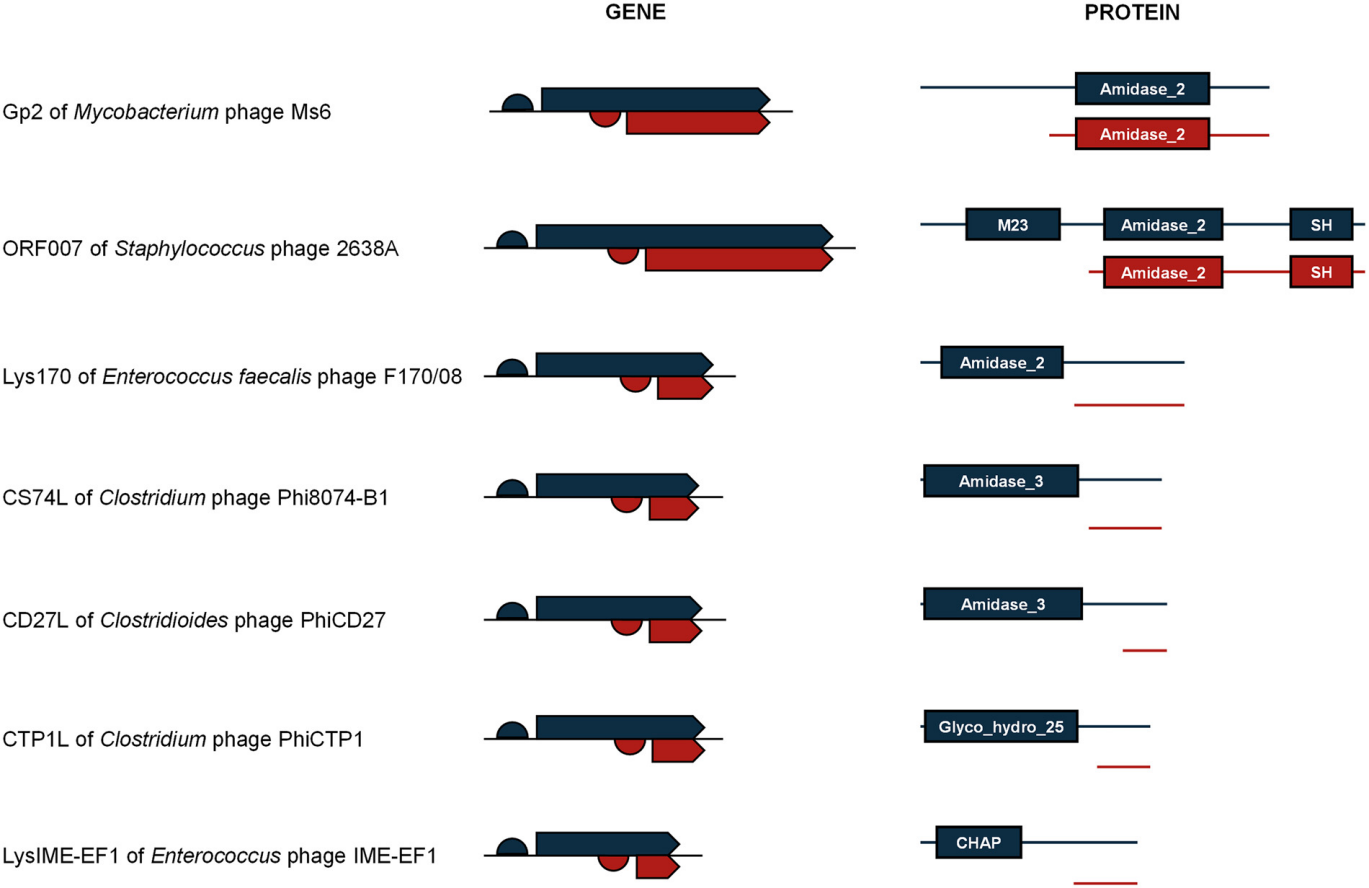
Known endolysins encoded in genes with two in-frame overlapping ORFs. In the endolysin genes the two in-frame overlapping ORFs are depicted by arrows; the blue and red ORFs encode the FLP and CTP, respectively. RBSs are represented by half circles. The conserved domains identified by Pfam in the FLP (blue) and CTP (red) are represented for each endolysin. Note that the putative or known C-terminal CWBD is not identified by the tool, except for ORF007. Amidase_2 (PF01510); M23, Peptidase_M23 (PF01551); SH, bacterial SH3_5 domain (PF08460); Amidase_3 (PF01520); Glyco_hydro_25 (PF01183); CHAP (PF05257).

Endolysins targeting Gram-positive bacteria and mycobacteria have a typical modular domain architecture, carrying one or more N-terminal catalytic domains (CDs) responsible for peptidoglycan cleavage and a domain at the C terminus involved in cell wall binding (17). The CDs can have different specificities, i.e., they can cut different chemical bonds on the peptidoglycan macromolecule (17). For two of the seven endolysins known to be encoded by two in-frame overlapping ORFs, Gp2 (LysA) of mycobacteriophage Ms6 and ORF007 of Staphylococcus phage 2638A (9, 10), the longest ORF leads to the synthesis of a full-length product (FLP) containing one or two identifiable CDs of different specificity, followed by a (putative or known) cell wall binding domain (CWBD). The shortest ORF originates a C-terminal product (CTP) carrying one CD and the CWBD (Fig. 1). For the other five endolysins, from enterococcal and clostridial phages (7, 8, 11), the FLP contains a single CD and one putative CWBD, while the CTP harbors solely the CWBD (Fig. 1).

For these five enterococcal and clostridial phage endolysins, interaction between the FLP and the CTP has been demonstrated (7, 8, 11). Lys170 and LysIME-EF1 were shown to exist as a heterotetramer made of one FLP and three CTP subunits, with the interacting interfaces lying in the CWBD moiety of each subunit, forming a tetrameric ring (7, 11, 18). The predominant species of CS74L, CD27L, and CTP1L have been proposed to be heterotetramers formed by two FLPs and two CTPs, with interactions through CWBDs also forming a tetrameric ring-like structure (8). Since multimerization of the FLP/CTP subunits increases the number of CWBDs in the holoenzymes, it was proposed that it provided a mechanism to enhance endolysin binding to the bacterial cell wall, and consequently potentiate lytic activity (7).

Here, we started with a bioinformatic analysis to find endolysin genes with two in-frame overlapping ORFs, by searching putative in-frame iTSSs in a sample of over 3,000 sequences. Afterward, we selected a few examples and investigated if two polypeptides were indeed produced from these genes, if the two polypeptides interacted to form a multimer, and how this impacted endolysin’s activity. Finally, we present the configuration of a new endolysin protein complex.

## RESULTS

### Endolysin genes with in-frame iTSSs are common in phages infecting Gram-positive bacteria.

**FIG 2:**
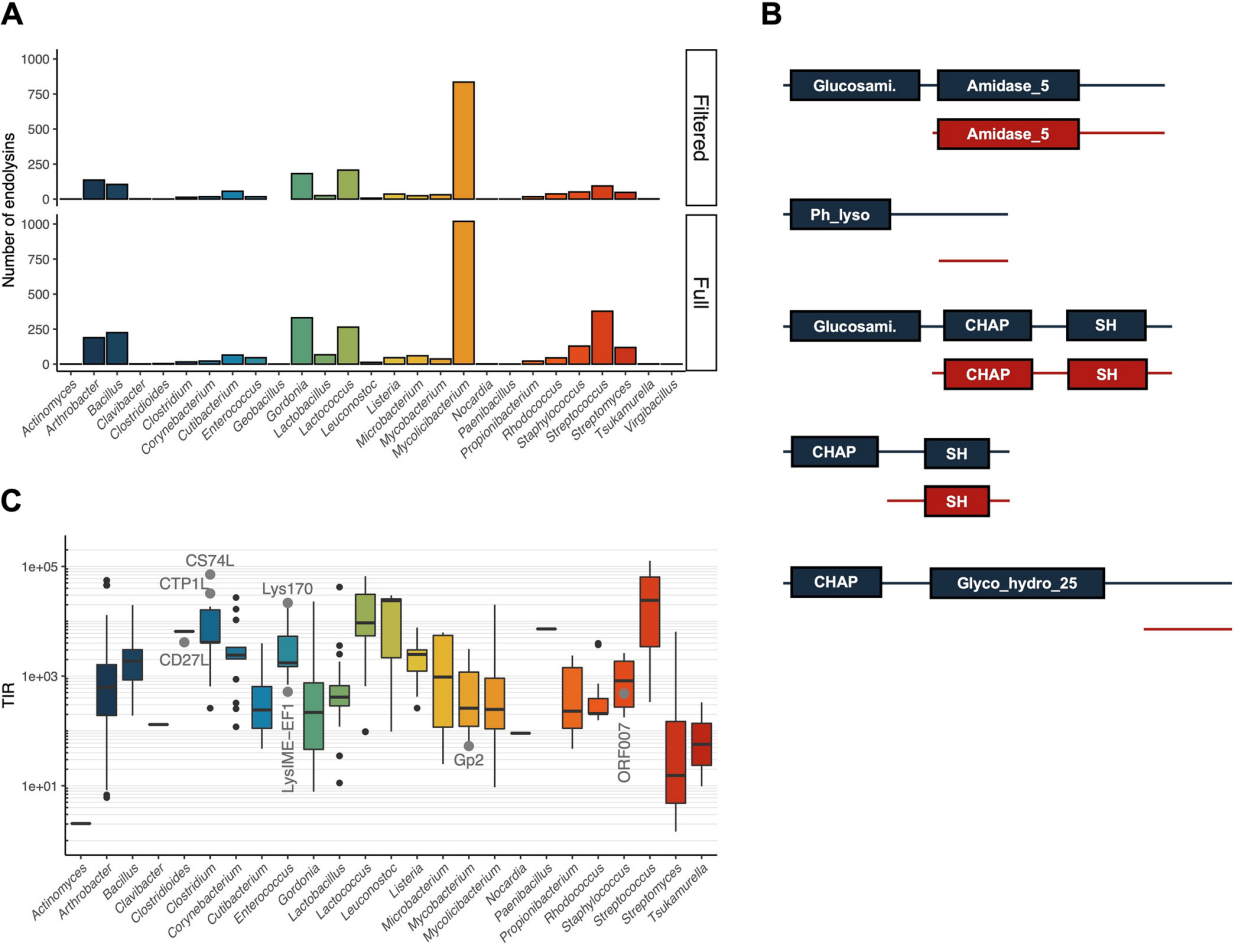
Identification of putative iTSS in endolysin genes. (A) Bar plot of the number of endolysins with predicted iTSS produced by phages infecting the bacterial genera indicated in the horizontal axis, in the initial sample (Full) and after the filtering steps (Filtered). The color code by genus is the same in panels A and C to facilitate comparisons. (B) Examples of different domain architectures represented in our filtered sample. Glucosami., N-acetylglucosaminidase (PF01832); Amidase_5 (PF05382); Ph_lyso, Phage lysozyme (PF00959); CHAP (PF05257); SH, bacterial SH3_5 domain (PF08460); Glyco_hydro_25 (PF01183). (C) Box plot showing the distribution of TIR values predicted for the iTSSs identified in endolysins of phages infecting bacteria of each genus. The color code by genus is the same in panels A and C to facilitate comparisons. With large gray circles are identified the TIRs from the confirmed iTSSs of endolysins encoded in genes with two in-frame overlapping ORFs (7–11). The small black circles correspond to outliers.

To investigate how widespread is the occurrence of endolysin genes with two in-frame overlapping ORFs, we derived the strategy schematically represented in Fig. S1 (supplemental material). We focused on the 3,780 endolysins of phages that infect Gram-positive bacteria and mycobacteria present on April 2020 in PhaLP (19), the reference database for phage lytic enzymes (Table S1 in the supplemental material). We chose to focus on this particular group of bacteria not only because of the previously described examples (7–11), but also because the large majority of endolysins from phages infecting Gram-negative bacteria have a single, globular domain corresponding to the CD (17), thus making unlikely the occurrence of iTSSs. From PhaLP, we extracted endolysin coding and AA sequences as well as information regarding the corresponding phage host. The mRNA sequences inferred from the endolysin coding sequences were submitted to RBS calculator (20) for prediction of putative in-frame iTSSs. A 4-step sequential filtering allowed us to select endolysins whose predicted in-frame iTSS with the highest translation initiation rate (TIR) had the following features: (i) a TIR value clearly above background for that sequence (see methods), suggesting a high likelihood of a meaningful iTSS; (ii) a localization not too close to the extremities of the gene, because if in the 5′ end it could result from misannotation of the gene start, as it is known to happen for several genes in the databases (21), and if in the 3′ end it would imply the production of a peptide smaller than the smallest conserved domain (the average length of the smallest CWBD [PF01473] is over 18 AA); (iii) a localization not inside conserved domain sequences since it would generate partial domains whose stability and functionality would be unlikely; and (iv) absence of phage tail domains, as they are suggestive of virion-associated lysins rather than endolysins, and correspond probably to misannotations. The 1946 endolysins that passed this filtering were ranked according to their maximum TIR score (Table S2). We have compared the initial sample of all endolysins of Gram-positive phages present in PhaLP with the filtered sample in terms of the taxonomical distribution of hosts (Fig. 2A). The results suggested that our filtering has not generated a taxonomical bias. Among the endolysins having iTSSs with high predicted TIR, we were able to identify domain architectures different from those described before (Fig. 2B).

We have performed additional analyses to help us increase the confidence in our in-frame iTSS predictions. (i) Based on a multiple sequence alignment, we built a percent identity matrix and clustered the AA sequences with over 50% identity. This clustering provided us with an additional empirical criterium to evaluate putative iTSSs, as if they appeared in the majority of the cluster members and always in the same relative position, we would be more confident of the predictions (see methods). (ii) We have analyzed another phage protein family (PF04860, portal proteins), in which in-frame iTSSs have never been described (Table S3). Contrary to the case of endolysins, where a putative in-frame iTSS was found in 51.5% of the genes, only 15.2% of those of portal proteins were predicted to have one. Of course, if we assume that iTSSs are absent from portal protein genes, which is consistent with the lack of description of this phenomenon in the literature, then these results suggest that our analysis can also generate some false positives and advises in favor of the experimental confirmation of the predictions. (iii) Finally, we have performed clustering at 90% AA identity (Table S4) to consider only nonredundant endolysin sequences and noted that among 635 unique sequences, 312 had a predicted in-frame iTSS (49.1%), confirming a prevalence of ~50% of endolysins with putative in-frame iTSSs among our universe of analyzed proteins.

One aspect of this analysis that has become apparent to us was that the predicted iTSS TIR varied vastly among host genera. RBS calculator (20) determines TIRs in relative units, and the determination is based on the free energies of key molecular interactions involved in translation initiation (22). Fig. 2C shows the box plots of the distribution of the TIRs by host genera, where it is possible to see that some genus have putative iTSSs with higher predicted TIRs than others (compare, e.g., Streptococcus with *Tsukamurella*) and that it did not correlate with how frequently iTSS have been identified in that genus (compare Fig. 2A with 2C). For comparison, we have determined the TIR for the experimentally verified in-frame iTSSs (7–11) (Table S5) and annotated them on the plot (Fig. 2C), which shows that even lower TIRs can be biologically relevant, in the sense that the corresponding iTSSs drive translation of the CTP. Note that the iTSS of Gp2 (LysA) of mycobacteriophage Ms6 has been experimentally confirmed (10) but has a TIR of only 53 while that of CS74L of the *Clostridium* phage Phi8074-B1, also experimentally verified (8), has a TIR of 71,221. Hence, while iTSSs with low TIRs cannot be disregarded, those with high TIR will have, in principle, higher probability of being real.

### Two polypeptides are produced from endolysin genes predicted to have an in-frame iTSS.

**(i) Selection of endolysins to address experimentally.** We chose four endolysins to experimentally test the bioinformatically predicted iTSSs, based on five criteria: (i) the cognate phage host genus should be medically or industrially relevant; (ii) we should have access to the host for downstream lytic activity assays; (iii) the endolysins should have varied domain architectures; (iv) the iTSSs should be at different relative positions in the sequence; and (v) the predicted TIR of the iTSS should be high.

Hence, we selected endolysins LysLW32 of Lactococcus lactis phage LW32, LysPollywog of M. smegmatis phage Pollywog, LysJavan488 of Streptococcus pyogenes phage Javan488, and LysP7951 of S. thermophilus phage P7951 (UniProt Acc. No. A0A1W6JHS3, A0A411CAU8, A0A4D6BBK1, and A0A286QQJ8, respectively). The iTSSs of these endolysins had high predicted TIRs, close to the maximum registered for their hosts genera (Fig. S2).

**FIG 3:**
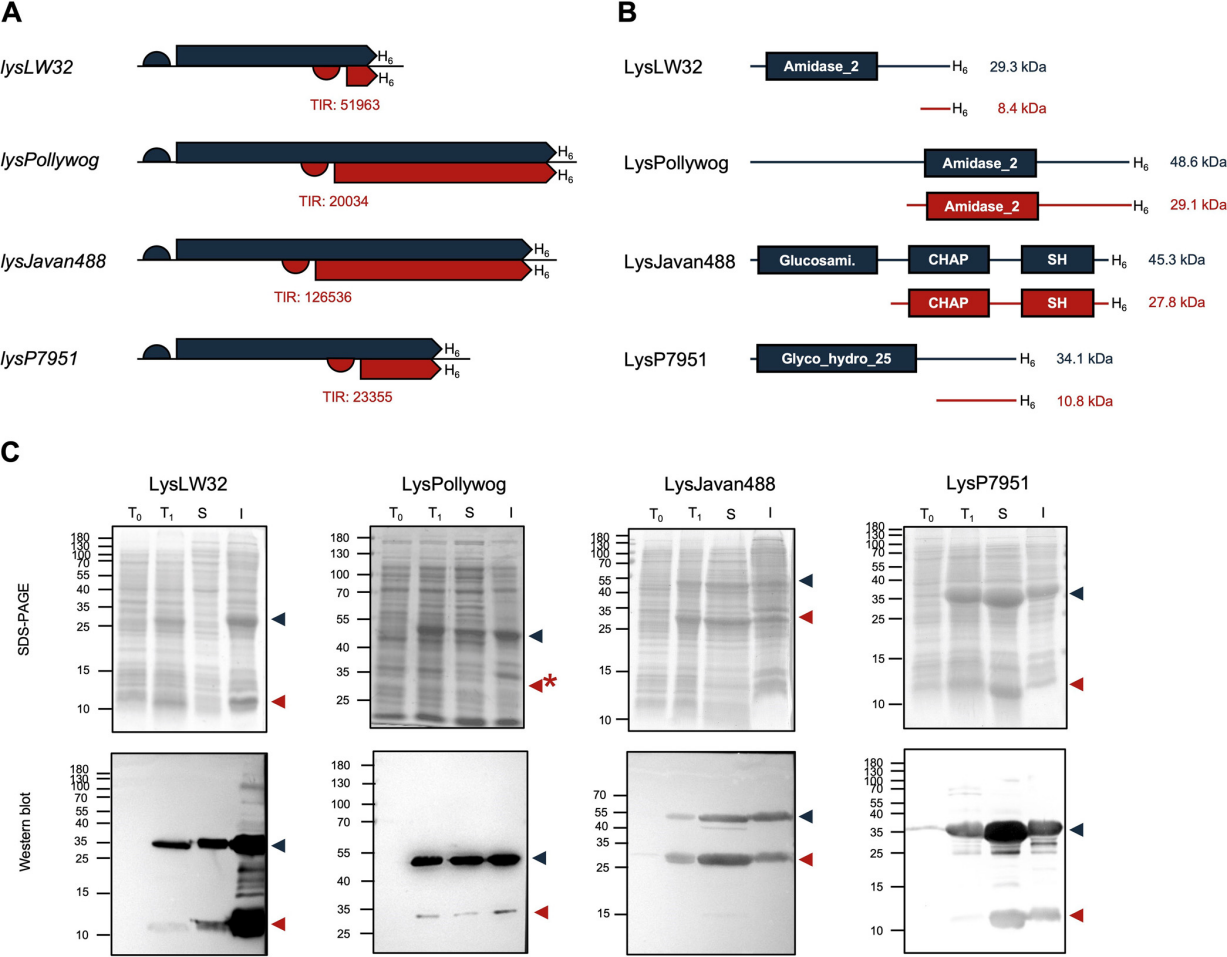
Endolysins with predicted iTSSs produce two polypeptides. (A) Schematic representation of the genes of the endolysins selected to address experimentally. RBSs are represented by half circles; CDS are represented by large arrows; hexahistidine tags are represented by H_6_; the TIR of the iTSSs are indicated below the CDS. (B) Schematic representation of the domain architecture of FLP and CTP of each selected endolysin. Amidase_2 (PF01510); Glucosami., N-acetylglucosaminidase (PF01832); CHAP (PF05257); SH, bacterial SH3_5 domain (PF08460); Glyco_hydro_25 (PF01183). (C) SDS-PAGE (top) and anti-His_6_ Western blots (bottom) of total protein extracts. T_0_, total protein extract before protein production induction; T_1_, total protein extract after protein production; S, soluble extract; I, insoluble extract. On the left side of each image is the molecular mass marker in kDa. The blue and red arrowheads indicate the FLP and CTP, respectively (the asterisk denotes a CTP that could not be unambiguously identified in the SDS-PAGE gel).

The relative position of the iTSSs and corresponding TIR, as well as the molecular masses and predicted conserved domains of FLP and CTP polypeptides are shown in Fig. 3A and B for each endolysin. Note that the indicated molecular masses include the hexahistidine (His_6_)-containing C-terminal tag PGGGS(H)_6_ used later for protein detection by Western blotting. Although the LysPollywog FLP has only one Amidase_2 domain identified by Pfam, the long N-terminal region suggests it carries an additional CD (Fig. 3B). The lack of identifiable functional domains is also notorious for the C terminus of three of the endolysins, where the CWBD is most often located.

**(ii) Protein production from endolysin genes with predicted in-frame iTSS.** The selected endolysin genes were cloned in an appropriate E. coli vector for generation of His_6_-tagged proteins. After induction of protein production, cell extracts were analyzed by SDS-PAGE and Western blot with an anti-His_6_ antibody. If the predicted iTSSs were acting as such, we would expect observing two new protein bands on the SDS-PAGE corresponding to the FLP and the CTP. Similarly, in this situation, two bands would be visible in the Western blot, given that the cloning added a His_6_ tag to the shared C terminus of both polypeptides (Fig. 3A and B).

In agreement with this, we were able to identify two overproduced polypeptides sharing the same C terminus from each endolysin gene, with electrophoretic mobilities compatible with the masses predicted for the corresponding FLPs and CTPs (Fig. 3B and C). With LysLW32 we could observe two protein bands in the total (T_1_) and insoluble extracts (I), compatible with the FLP and CTP masses. In the soluble extract (S) the two LysLW32 polypeptides were only detected in the Western blot. For LysPollywog, LysJavan488 and LysP7951, the FLP and CTP forms were detected in the total extract (T_1_), as well as in the soluble (S) and insoluble (I) fractions (Fig. 3C).

We would like to note that the amount of LysPollywog CTP (red arrowhead in Fig. 3C) produced from the iTSS was much lower than that of the FLP (blue arrowhead). This might reflect the phylogenetic distance between the expression host (E. coli) and the phage host (M. smegmatis), which entails differences in their Shine-Dalgarno sequences and mRNA GC content that might interfere with translation initiation from the iTSS. Note that the translation signals of the largest ORF are given by the expression vector.

In conclusion, we were able to experimentally confirm the bioinformatically predicted iTSSs in four endolysin genes, from phages infecting four different bacterial species and producing enzymes with distinct domain architectures.

### The two endolysin polypeptides do not always interact to generate an heteromeric enzyme.

With the confirmed iTSSs for the four endolysins we continued investigating if interaction between FLP and CTP polypeptides occurred, as described for five of the seven endolysins with demonstrated iTSS (see above). Hence, we proceeded with purification of the endolysins by affinity chromatography (AF) via their C-terminal His_6_-tags, which allowed us to purify both polypeptides regardless of their interaction. We have excluded LysLW32 from this analysis since we could not obtain soluble polypeptides in sufficient amount to proceed with the analysis (Fig. 3C).

We have obtained three distinct elution profiles from the AF: (i) LysPollywog eluted in one peak with a very small fronting tail (~1.5 mL) (Fig. S3A). Analysis of the AF fractions by SDS-PAGE (Fig. S3B) showed that both LysPollywog polypeptides composed the elution peak. (ii) For LysJavan488 we observed slow and prolonged protein elution during the first washing step (75 mM imidazole), before the complete elution of the remaining bound protein with 500 mM imidazole (Fig. S3A). SDS-PAGE analysis of fractions from the 75 mM imidazole elution revealed the two LysJavan488 polypeptides in comparable amounts, while the 500 mM imidazole peak contained a much larger quantity of the CTP (Fig. S3B). (iii) For LysP7951 we observed a major peak followed by a second “shoulder” peak (Fig. S3A). Here, the major peak was mainly composed by the FLP, whereas the second peak contained both polypeptides producing bands of similar intensity in SDS-PAGE (Fig. S3B).

**FIG 4:**
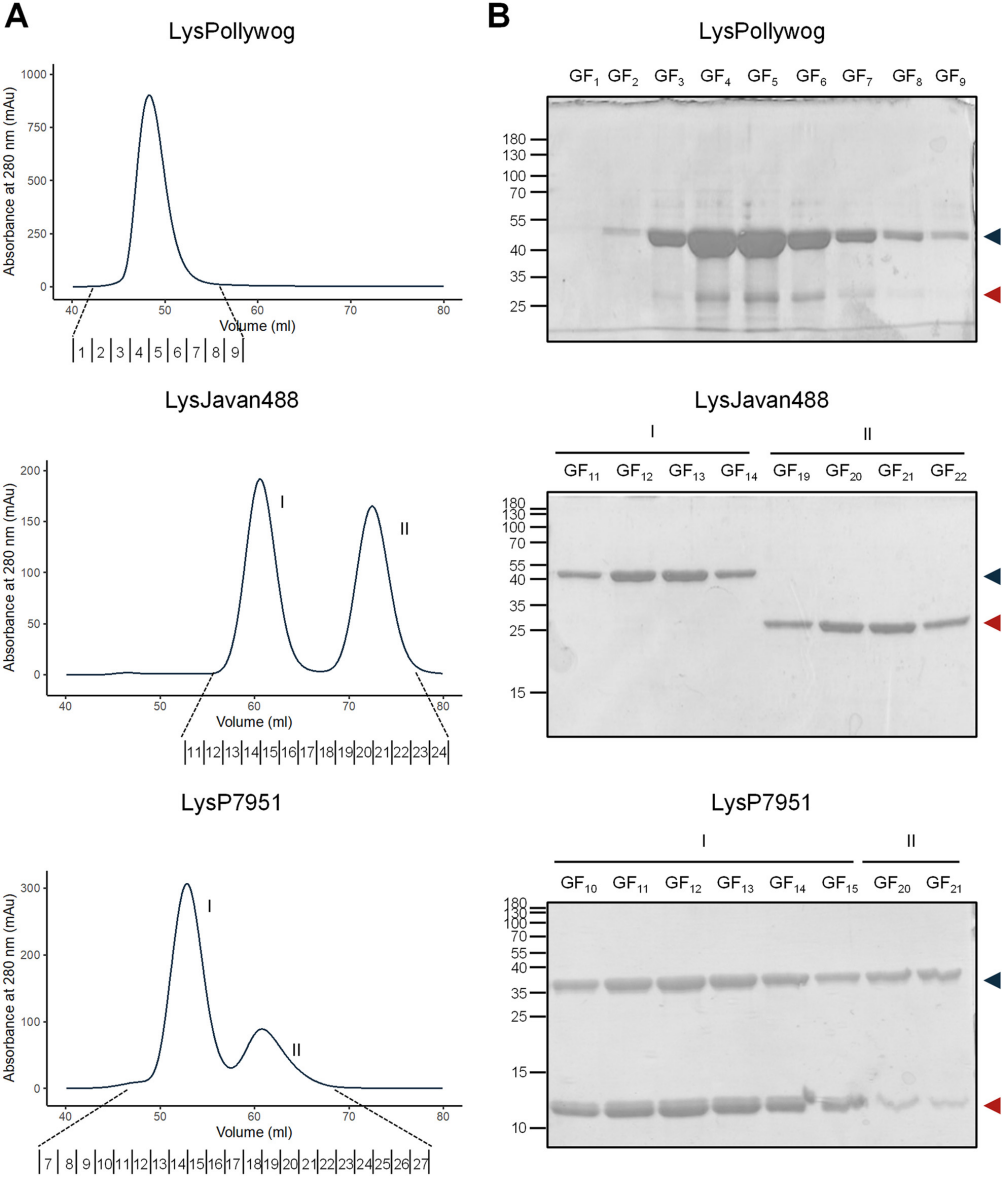
Study of FLP/CTP interaction by GF analysis. (A) Chromatogram showing the elution profile of the endolysins analyzed by GF. The numbers shown above the horizontal axis represent the collected fractions and correspond to those analyzed in panel B. (B) SDS-PAGE analysis of the GF fractions. The fraction numbers correspond to those shown in A. On the left side of each image is the molecular mass marker in kDa. The blue and red arrowheads indicate the FLP and CTP, respectively.

To investigate if FLP and CTP interacted to form a complex and eventually a heteromultimer, we subjected AF fractions, with as much as possible comparable amounts of each polypeptide, to gel filtration chromatography (GF). If the two polypeptides were monomeric and did not interact, they would elute from the GF column as two distinct peaks, according to their molecular masses. On the contrary, if they stably associated, the polypeptides would jointly elute as a major peak, in principle with an elution volume smaller than that of the isolated polypeptides. Fig. 4 shows the GF elution profiles of the three endolysins (Fig. 4A) alongside the SDS-PAGE analysis of the GF fractions (Fig. 4B). Interestingly, also here we observed three different behaviors. (i) For LysPollywog we saw that FLP and CTP eluted as a single peak close to the column’s void volume, set around 45 mL (see reference 7 for the calibration procedure of the column), which was an indication of large molecular mass species present in solution (with over 150 kDa). While this result was suggestive of FLP/CTP association, we note that in our protein preparation there was a much larger amount of the FLP compared to CTP, which advises against a definitive conclusion of interaction. (ii) The scenario was much clearer for LysJavan488 as FLP and CTP eluted separately as two independent peaks, indicating that the two polypeptides did not associate, at least under the conditions tested. (iii) Finally, for LysP7951 we observed a major peak followed by a small secondary peak: the major peak contained both polypeptides, with CTP detected with a slightly higher intensity than the FLP in SDS-PAGE, whereas the second peak contained a higher proportion of FLP. These observations suggested that the first peak could be composed of a complex formed by FLP and CTP polypeptides of LysP7951, probably a heteromultimer, while the second peak most likely corresponded to nonassociated and/or incompletely associated polypeptides.

In conclusion, the GF analysis indicated that the LysJavan488 polypeptides could not form stable associations, while those of LysP7951 did so, showing that the presence of iTSSs does not necessarily imply that FLP and CTP form a complex.

### Both LysJavan488 polypeptides are active against S. pyogenes.

In the previous section we have shown that the two products of *lysJavan488* did not associate. Next, we set up to understand if both polypeptides were active, since they shared the CHAP CD and the SH3_5 CWBD (Fig. 3B). To study this, we have evaluated the lytic action of each of the products of *lysJavan488* against S. pyogenes. Protein solutions prepared from GF fractions (Fig. 4B) were spotted on dense lawns of viable cells and the formation of halos resulting from cell lysis evaluated after overnight incubation. Although somewhat weak, halos were visible with 16 μM each polypeptide (Fig. S4) showing that both the CTP and the FLP were active. Hence, for LysJavan488, the products of its two ORFs do not associate in the tested conditions and are both active.

### Only the LysP7951 putative complex is active against S. thermophilus.

To investigate the lytic activity of the LysP7951 putative complex (first GF peak, Fig. 4) and of its individual subunits (FLP and CTP), we have generated two mutants: *lysP7951_209-310_*, corresponding solely to the smallest ORF and hence allowing us to purify the CTP as a single species, and *lysP7951_M209L_*, where the iTSS and corresponding RBS were eliminated and thus leading solely to the production of the FLP, also allowing its independent purification. As before, we have overproduced LysP7951_209-310_ and LysP7951_M209L_ and purified them by AF and GF (Fig. S5).

**FIG 5:**
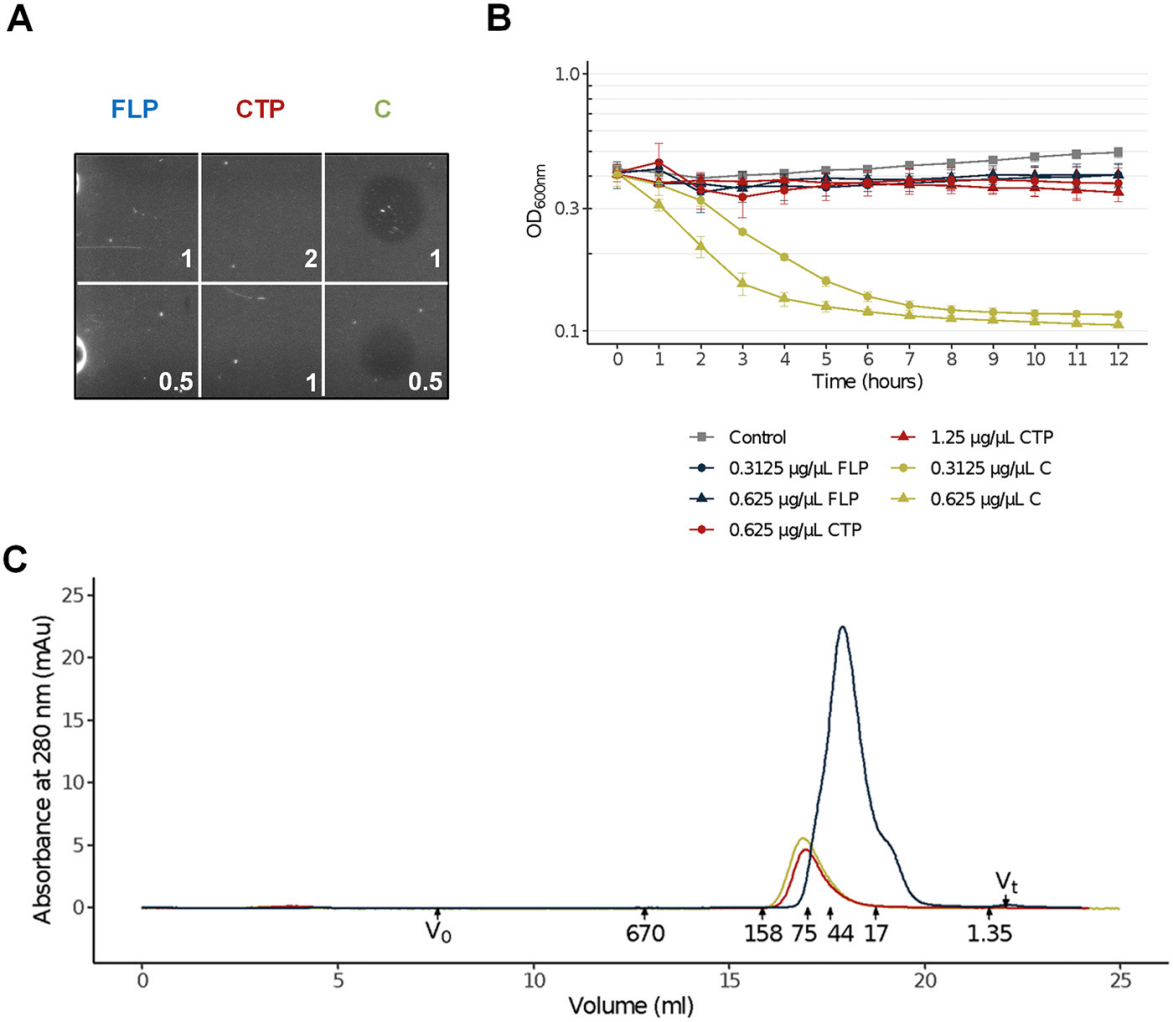
Lytic activity of the putative LysP7951 complex and its FLP and CTP isoforms. (A) Lytic activity of the LysP7951 putative complex (C) and of its putative subunits FLP (LysP7951_M209L_) and CTP (LysP7951_209-310_) was evaluated by spotting the indicated protein amounts (μg) on a dense lawn of S. thermophilus cells. (B) Lytic activity in liquid medium of the LysP7951 putative complex (green) and of the FLP (blue) and CTP (red) against S. thermophilus cells. Control cells, to which only protein buffer was added are shown in gray. Triangles correspond to the higher concentrations while circles correspond to lower concentrations. (C) Analytical GF chromatogram showing the elution profiles of LysP7951_M209L_ (FLP, in blue), LysP7951_209-310_ (CTP, in red) and of the LysP7951 putative complex (in green). Over the horizontal axis are indicated the elution volumes of the proteins of the standard (indicated with the corresponding kDa) alongside the total (V_t_) and void (V_0_) volumes.

The lytic activity of the LysP7951 putative complex and of the individual FLP and CTP polypeptides (LysP7951_M209L_ and LysP7951_209-310_, respectively) was tested by spotting them on a dense lawn of S. thermophilus cells. Here, we observed lysis halos with the LysP7951 putative complex, but not with the isolated FLP and CTP (Fig. 5A). For CTP this observation was to be expected given the absence of any CD. However, this is not true for FLP that contains the Glyco_hydro_25 CD, but it is in line with what has been reported for Lys170, whose FLP subunit showed poor lytic activity (7).

We also assayed lytic activity in liquid medium and made very similar observations: lytic activity, measured as the reduction in optical density at 600 nm (OD_600nm_) of a S. thermophilus cell suspension, was only observed with the LysP7951 putative complex with a dose-dependent response (Fig. 5B). Together, these results showed that the presence of the two LysP7951 isoforms (FLP and CTP) is required for lytic activity against S. thermophilus and highlights the importance of LysP7951 being encoded in a gene with two in-frame overlapping ORFs.

### LysP7951 FLP and CTP heteromultimerize.

We then set up to investigate the interaction between LysP7951 FLP and CTP isoforms and to demonstrate their association as a complex. For that, we have subjected the three protein preparations (LysP7951 putative complex, LysP7951_209-310_, and LysP7951_M209L_) to analytical GF in a previously calibrated column (Fig. 5C), and from the elution volumes of each protein species (16.89, 16.93 and 17.91 mL, respectively) we estimated their apparent molecular masses. The molecular mass calculated for the FLP (i.e., LysP7951_M209L_) was 34.6 or 35.0 kDa, if estimated directly from the molecular masses or the Stokes radius of the proteins of the standard, respectively (Fig. S6). This agreed with the molecular mass inferred from the primary sequence (34.1 kDa), suggesting that FLP was monomeric in solution. The same was not verified for the CTP (i.e., LysP7951_209-310_), for which the analogous estimation of the molecular mass was 73.7 and 75.7 kDa, respectively. Such a mass was compatible with a CTP homomultimer of seven subunits (7 × 10.8 = 75.6 kDa).

These observations suggested that in the context of the LysP7951 complex the CTP could be participating with up to six subunits, the seventh being provided by the FLP, which was probably present as a single subunit in the putative complex. Additionally, the absorbance of the same mass of each protein species was very different, with that of FLP being much higher than those of the CTP and LysP7951 putative complex (Fig. 5C). The predicted molar extinction coefficient of the two individual polypeptides is very distinct (53080 M^−1^cm^−1^ for LysP7951_M209L_ and 2980 M^−1^cm^−1^ for LysP7951_209-310_), and while this might be interfering with the registered absorbance, the lower absorbance of the LysP7951 putative complex also suggested it was formed by a higher proportion of CTP subunits relatively to those of FLP.

The putative LysP7951 heteromultimer eluted with an apparent molecular mass of 76.0 and 78.1 kDa, respectively. Such a molecular mass would be compatible with an heteromultimer containing one FLP coupled to four CTP subunits (34.1 + 4 × 10.8 = 77.3 kDa).

### Active LysP7951 complex can be reconstituted from the FLP and CTP polypeptides.

**FIG 6:**
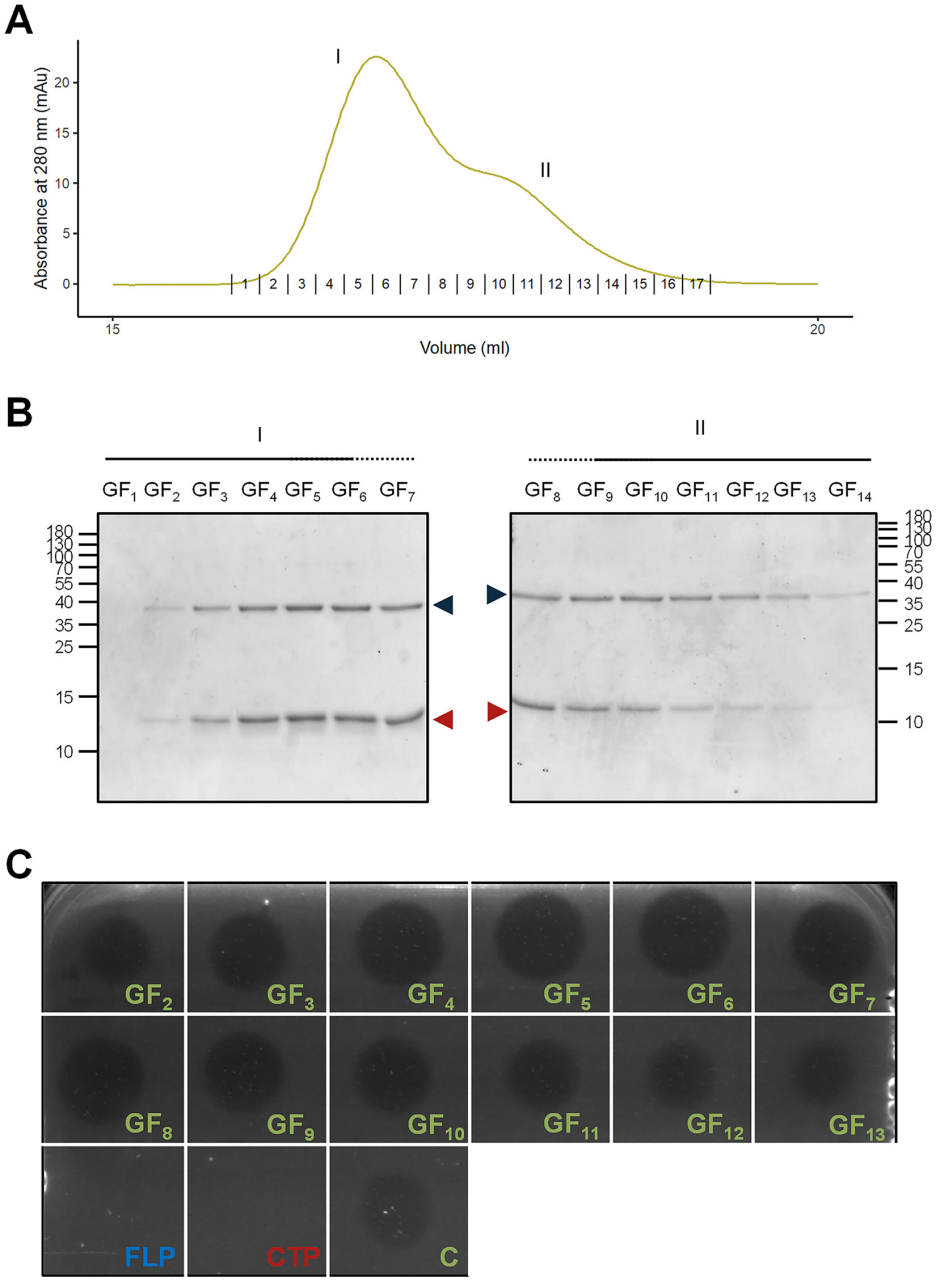
Reconstitution of the LysP7951 complex and its lytic activity against S. thermophilus cells. (A) Analytical GF chromatogram showing the elution profile of the LysP7951 heteromultimer reconstituted from independently purified FLP and CTP. The numbers shown above the horizontal axis represent the collected fractions and correspond to those analyzed in panel C. (B) SDS-PAGE analysis of samples of the fractions indicated in panel A. On the side of each image is the molecular mass marker in kDa. The blue and red arrowheads indicate the FLP and CTP, respectively. (C) Activity of the same volume of the GF fractions of the reconstituted heteromultimer shown in panels A and B. The GF fraction numbers correspond to those indicated in panels A and B. FLP (1 μg), CTP (2 μg) and the LysP7951 complex (C, 1 μg) were spotted as controls.

Based on the analytical GF, the Lys7951 complex could be formed by one FLP associated with up to six CTPs. Thus, we investigated if we could reconstitute the active LysP7951 heteromultimer by mixing the individually purified LysP7951_M209L_ and LysP7951_209-310_. Hence, we have incubated LysP7951_M209L_ and LysP7951_209-310_ at a 1:6 molar ratio and subjected the mixture to analytical GF. The protein mixture eluted as a single peak with a small right “shoulder,” with the peak elution volume being very close to that of the LysP7951 complex (16.87 vs.16.89 mL, Fig. 6A and 5C). This supported that the endolysin heteromultimer was reconstituted through LysP7951_M209L_ and LysP7951_209-310_ association. The SDS-PAGE analysis revealed that the two protein species peaked in the same fractions (GF_5_/GF_6_) and that the peak shoulder contained an excess of LysP7951_M209L_ over LysP7951_209-310_, suggestive of incomplete heteromultimerization (Fig. 6B).

We have then investigated the lytic activity of the fractions obtained from the analytical GF. The size of the obtained lysis halos correlated with the amount of heteromultimer present in each fraction (compare Fig. 6B and C), with the peak fractions GF_5_ and GF_6_ originating the largest halos. Again, this supported the hypothesis that the heteromultimer is the active form of LysP7951 and that its activity can be reconstituted by combining the individual subunits.

In conclusion, we have shown that only the LysP7951 complex, and not the individual polypeptides, is active and that both the heteromultimer and the activity can be reconstituted by mixing the FLP and CTP endolysin subunits.

### Investigation of the composition of LysP7951 complex by MS and SAXS.

We have performed mass spectrometry (MS) and small-angle X-ray scattering (SAXS) studies to have a more precise view of the LysP7951 complex composition. MS analysis of FLP (LysP7951_M209L_) and CTP (LysP7951_209-310_) in denatured conditions confirmed their predicted molecular masses when monomeric. The spectrum in Fig. S7A shows the charge-state distribution (CSD) generated by the FLP from +30 to +15 charges and an experimental mass of 34,063.70 Da, which deviates 0.58 Da from the sequence calculated monoisotopic mass (34,064.28 Da). The CTP (Fig. S7B) generated a CSD from +13 to +6 charges and the experimental mass was 10,790.40 Da, with a deviation of 0.20 Da from the theoretical mass (10,790.20 Da).

**FIG 7:**
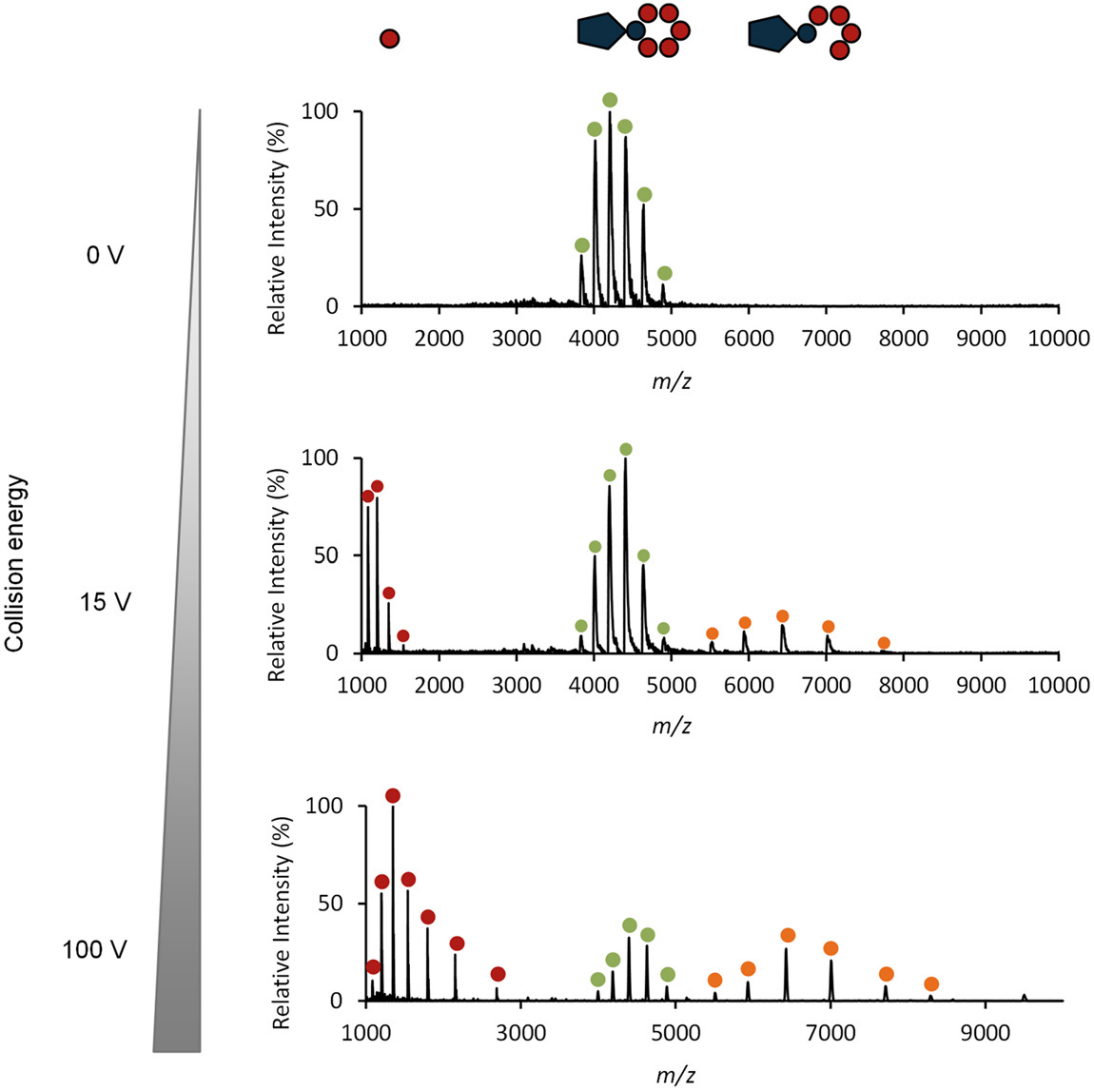
Native mass spectrometry analysis of LysP7951 protein species. The analysis shows the effect of collision energy on the complex quaternary structure. In the first spectrum (0 V) only one CSD is present at *m/z* 3,900–5,000 with +23 to +18 charges, corresponding to a mass of 88,229 ± 92 Da, which is compatible with 1 FLP noncovalently interacting with 5 CTP subunits. When the collision energy is increased to 15 V, one CTP subunit is expelled from the complex and two more CSD are generated: one at *m/z* 1,000–2,000 corresponding to the CTP (red dots) and a mass of 10,808 Da, and the other at *m/z* 5,500–8,000 generated by the complex with only 4 CTP subunits (orange dots) with 77,260 Da. The complex with 5 CTP subunits is still visible and is the main form in solution (green dots). Collision energy was further increased to 100 V, and the CSD observed in the spectrum for 15 V are still present, but the intensities of the 1:4 complex and that of the CTP are higher, while that of the 1:5 complex diminishes.

Native MS was used to investigate the stoichiometry of the complex formed between FLP and CTP. The LysP7951 complex generated a CSD comprehended between *m/z* ~3,900 and 5,000 with +23 to +18 charges (Fig. 7, top spectrum), corresponding to a mass of 88,229 Da. This value was compatible with a 1:5 stoichiometry of the FLP and CTP subunits (34.1 + 5 × 10.8 = 88.1 kDa) and agreed with the analytical GF data that suggested a 1:4 to 1:6 stoichiometry. Increasing the collision energy led to complex dissociation in the gas phase (Fig. 7). For a collision energy of 15 V, three CSD were present in the spectrum corresponding to the ~88 kDa complex (green dots), the ~10.8 kDa CTP (red dots) and the complex with only 4 CTP subunits (~77 kDa, orange dots). When the collision energy was increased to 100 V, the same CSD was observed, but the intensities of the 1:4 complex as well as of the CTP increased, whereas the intensity of the 1:5 complex decreased.

**FIG 8:**
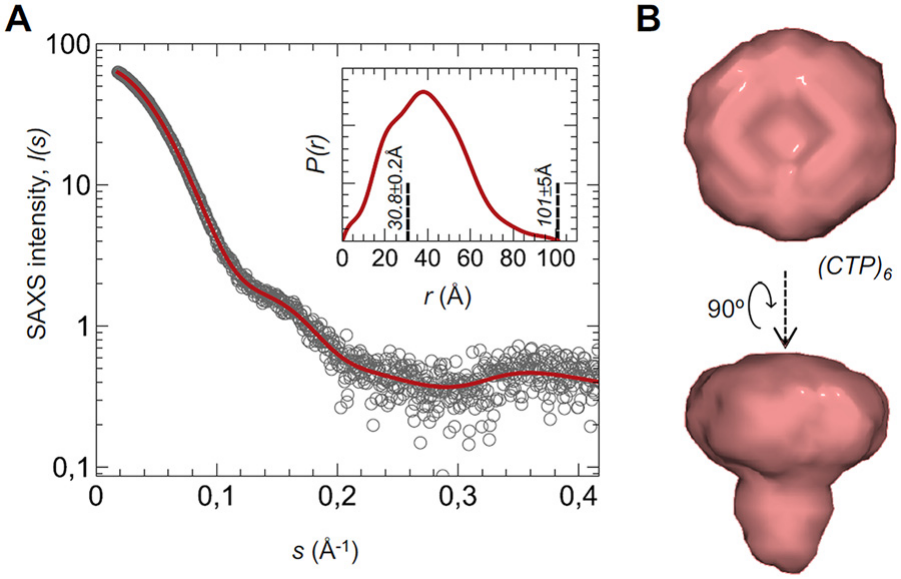
SAXS analysis of the LysP7951 CTP subunit homomultimer. (A) SAXS intensity of LysP7951 CTP oligomer (circles), *I(s)*, is represented in logarithmic scale as a function of the momentum of transfer, *s*. The red line corresponds to the scattering profile calculated from the *ab initio* model that best fitted the experimental data (χ^2^ = 1.07). The inset shows the pairwise distance distribution, *P(r)*, of SEC-purified CTP calculated from data in the range 0.0182 < *s* < 0.417 Å^−1^. The derived *Rg* and *Dmax* values are displayed in dashed lines. (B) SAXS-generated *ab initio* molecular envelope obtained by clustering and averaging models from 20 independent runs, with a Normalized Spatial Discrepancy (NSD) of 1.26 Å.

A 1FLP:5CTP stoichiometry implied that six CTP moieties were present in the LysP7951 complex. Thus, one would expect the purified CTP homomultimer to be a hexamer of 64.8 kDa (6 × 10.8 kDa) in solution, despite its apparent mass in the analytical GF being compatible with up to a heptamer. Unfortunately, native MS failed to give reliable measurements of the CTP homomultimer mass, and therefore we attempted to estimate it by SAXS. The molecular mass estimation from the SAXS data (23) for LysP7951 CTP was 69.9 kDa, in line with a putative homo-oligomer comprised of 6–7 subunits. The SAXS data disclosed an oligomeric assembly in solution with a radius of gyration (*Rg*) of 30.75 ± 0.20 Å and a maximum distance (*Dmax*) of 101 ± 5 (Fig. 8A). The bimodal and asymmetrical pairwise distance distribution, *P(r)*, indicates that the oligomer is nonglobular. The corresponding SAXS-driven *ab initio* reconstruction assuming a hexamer state (Fig. 8B) highlights a disk-like shape with a slight central depression and protrusion on the opposite side, with an overall silhouette resembling a mushroom that is in excellent agreement with the experimental data (χ^2^ = 1.07).

Altogether, the MS and SAXS analysis indicated that the LysP7951 enzymatic complex corresponds to a 1FLP:5CTP hetero-multimer, a stoichiometry never observed before in phage endolysins.

## DISCUSSION

This study aimed to investigate the occurrence of phage endolysins that are produced as two isoforms, by genes with an in-frame iTSS, and how this impacts endolysin activity. As mentioned in the introductory section, endolysins are building a momentum as alternative antimicrobials to treat antibiotic resistant infections (15, 16), being thus of great relevance understanding their structure-function interplay. Our approach allowed us to first identify endolysin genes with putative iTSSs in phages that infect Gram-positive bacteria. We have then selected a few examples and experimentally confirmed that two polypeptides were indeed produced from genes with predicted iTSSs, but that not always does the association between the polypeptides occurs.

Our bioinformatic analysis supported the identification of endolysin genes with iTSSs in phages infecting all analyzed bacterial genera, apart from *Geobacillus* and *Virgibacillus* (Fig. 2A). At this point we should be careful with the interpretation of this result as it might be simply due to the low number of endolysin sequences of phages infecting these two genera available in the initial sample. Still, what is striking is that endolysin genes with in-frame iTSSs appear to be quite common and not just an exception of a few phages (7–11), something which has eluded us until now.

Our experimental analysis confirmed the presence of iTSSs in four endolysin genes, from phages infecting bacteria from different genera and whose products had distinct domain architectures and catalytic specificities. The studied endolysins with now known iTSSs can be broadly divided in two groups. In one group the lytic enzymes have two CDs and one CWBD, with the iTSS located between the CDs, which leads to the production of a smaller polypeptide containing the second CD and the CWBD. Included here are the endolysins of mycobacteriophages Ms6 (10) and Pollywog (this work), of the staphylococcal phage 2638A (9), and of the streptococcal phage Javan 488 (this work). The other group comprises the endolysins carrying only one CD and one CWBD, with the iTSS located between these two domains, which leads to the production of a smaller polypeptide containing only the CWBD. These are the endolysins of enterococcal phages F170/08 and IME-EF1 (11), of clostridia phages Phi8074-B1, phiCTP1, and PhiCD27 (8), of L. lactis phage LW32, and of S. thermophilus phage P7951 (this work). Interestingly, heteromultimerization of the two polypeptides has only been describe for endolysins of the second group, although it is not clear if interaction between the two polypeptides of Gp2 (LysA) of M. smegmatis phage Ms6 and ORF007 of Staphylococcus phage 2638A was never observed or simply never tested. Even from our examples, interaction was demonstrated for LysP7951 (from the second group), not observed in LysJavan488 (first group), but inconclusive for LysPollywog. Further investigation on this aspect will certainly be of interest.

While heteromultimerization of the two polypeptides produced from endolysin genes with two in-frame overlapping ORFs has already been demonstrated for six endolysins, including here for LysP7951, it is worth noticing that three different stoichiometries have been reported. For the enterococcal endolysins Lys170 and LysIME-EF1, the observed stoichiometry was 1FLP:3CTP, which generates endolysins with one CD and four CWBDs (one from the FLP and three from the CTP subunits) (7, 11). For the clostridia endolysins CS74L, CD27L and CTP1L, a dimer of a FLP:CTP heterodimer was proposed as the predominant stoichiometry, which generates an endolysin with two CDs and four CWBDs (two from the FLPs and two from the CTPs) (8). Our data regarding the streptococcal LysP7951 indicates it displays yet another stoichiometry, with the enzymatic complex exhibiting a 1FLP:5CTP configuration, leading to the assembly of an endolysin with one CD and six CWBDs (five from the CTPs and one from the FLP). We have excluded for LysP7951 the 2FLP:2CTP stoichiometry proposed for the clostridia endolysins based on three major observations: (i) the much lower absorbance of the LysP7951 complex compared to the isolated FLP (LysP7951_M209L_), which indicated a FLP:CTP ratio <1 in the complex (Fig. 5C); (ii) the estimated molecular mass of the isolated CTP in the analytical GF and SAXS (around 70 kDa), which strongly pointed for a multimeric state higher than the 4-mer; and (iii) the mass of the complex precisely determined by native MS (88.2 kDa), which was closer to that expected for the 1FLP:5CTP configuration (88.1 kDa) than for the 2FLP:2CTP architecture (2 × 34.1 + 2 × 10.8 = 89.8 kDa). To the best of our knowledge this is the first time that such a 1:5 stoichiometry is reported for an heteromeric endolysin.

While the importance of the iTSS and of the two produced polypeptides is somewhat obvious from their role in heteromultimerization, it was still very interesting to observe that lytic activity against S. thermophilus could only be detected with the LysP7951 multimer. Note that the FLP subunit contains the CD and the CWBD and yet activity could not be observed even with immobilized cells and 1 μg of the protein (~0.03 nmol) (Fig. 5A). This is in line with what has been observed before for Lys170 (7), in which activity of the corresponding FLP subunit against immobilized E. faecalis cells was only observed with 10 times more protein (~0.3 nmol), and even so much weaker than with the Lys170 heteromultimer. As discussed previously, multimerization could have evolved as a strategy to increase the number of CWBDs, providing the enzymatic complex with the necessary avidity for efficient binding to the cell wall and subsequent cleavage activity (7).

The role of both endolysin gene products under the situation in which interaction is not observed is somewhat less intuitive. Here, for LysJavan488 we observed that both FLP and CTP were active. In the case of ORF007 of Staphylococcus phage 2638A, whose iTSS is also located between the two CDs, activity in analogous spot lysis assays also showed that both polypeptides were active (9). The combined action of the two polypeptides can have relevance for other aspects of endolysin activity that have not been evaluated here. In fact, the action of both polypeptides of Gp2 (LysA) of mycobacteriophage Ms6 was shown to be important for proper lysis timing of infected host cells (10).

In conclusion, here we have shown that the existence of endolysin genes with two in-frame overlapping ORFs is not just an eccentricity of a few endolysins, but it is a widespread phenomenon in this protein family. Moreover, we show that not in all cases is multimerization of the two produced polypeptides the final goal but found one streptococcal endolysin that heteromultimerizes with a stoichiometry never reported before for multimeric endolysins. This work not only brings forward a neglected aspect of endolysin biology with possible important repercussions for their applicability as enzybiotics, as it raises several questions that are worthy of future research efforts.

## MATERIALS AND METHODS

### Identification of potential iTSSs.

We gathered the information available in PhaLP database (19) concerning endolysins of phages infecting Gram-positive bacteria. On April 2020, 3,780 such endolysins were listed and information regarding their DNA and AA sequences, and phage host have been retrieved (Table S1). Multiple sequence alignments of the endolysin AA sequences were generated with MAFFT (24) with the settings set to auto. Clustering of endolysins based on AA sequence identity was performed using custom python scripts described and available elsewhere (25). Conserved protein domain analysis was performed with a local installation of HHMER version 3.1b2 (26) to search PfamA version 31.0 profiles (27). Coding sequences were converted to mRNA sequences using a custom python script and submitted to RBS calculator (20) for identification of putative iTSSs. The setting “organism” was defined as the phage host, which corresponds to the organism in which translation initiation at the iTSS will occur in a natural context. When not available, the closest available relative (i.e., same genus) was selected. For each sequence, the in-frame (frame 0 in RBS calculator) iTSS with the highest score was registered and the domain architecture and position of the putative iTSS mapped in each sequence of each cluster (Fig. S9, custom python and R scripts, respectively).

Next, a 4-step filtering was performed (custom python script and Table S2): (i) iTSSs that were not statistical outliers in the sequence, i.e., iTSSs whose RBS calculator predicted TIR (translation initiation rate) was not clearly above background for that sequence led to the exclusion of the endolysin; (ii) iTSSs in the extremities of endolysin genes (within the sequences coding for the first or last 20 AA) also led to the exclusion of the endolysin, to account for possible cases of misannotation of the gene start or whose gene product would be too small to accommodate even small, conserved protein domains; (iii) iTSSs located inside conserved protein domains also led to the exclusion of the endolysin, since they would generate partial domains; and (iv) proteins containing phage tail domains were excluded as they are most likely misannotated virion associated lysins and not endolysins.

To test the robustness of our analysis, a similar protocol was performed for another phage protein family for which cases of genes with overlapping in-frame ORFs were never described. For this analysis we used portal proteins of phages infecting the same hosts analyzed in the endolysin screen and available at Pfam under PF04860 (Table S3).

### Construction of endolysin expression strains.

LysLW32 and LysPollywog coding genes were commercially synthesized (NZYTech, Portugal) (Table S6). LysJavan488 and LysP7951 coding genes were amplified by PCR from the genome of the respective phages with specific primers carrying NcoI and XmaI recognition sites (Table S7). Gene *lysJavan488* was amplified from the genome of the Javan488 prophage on the genome of S. pyogenes M1 GAS and *lysP7951* from the S. thermophilus phage P7951. LysP7951_209-310_ coding sequence was obtained through PCR using *lysP7951* as the template and one additional internal primer (Table S7). LysP7951_M209L_ coding sequence, in which the internal RBS and starting codon of *lysP7951* were eliminated, was obtained through overlap PCR with overlapping primers carrying the mutations (Table S7). All genes were cloned into pIVEX2.3d (Roche Applied Sciences) via NcoI and XmaI for construction of C-terminal His_6_-tagged proteins. The ligation mixtures were transformed into chemically competent Escherichia coli XL1-Blue MRF’ (Stratagene) prepared as described elsewhere (28). Selection was made at 37°C in LB medium supplemented with 100 μg/mL Ampicillin. The plasmids were screened by PCR with vector-specific primers (Table S7), recovered, sequenced and transformed into chemically competent cells (28) of the expression E. coli strain CG61. This strain is a BL21 derivative that produces phage T7 RNA polymerase upon thermal induction (29). Selection was made at 28°C in LB medium supplemented with 40 μg/mL Kanamycin and 100 μg/mL Ampicillin.

### Induction and evaluation of endolysin production.

Expression strains were grown overnight at 28°C in LB medium supplemented with selecting antibiotics, and with aeration. Cultures were diluted 100-fold in LB (for the strain overproducing LysP7951) or buffered (0.1 M sodium phosphate buffer pH 7) LB supplemented with 0.5 M D-sorbitol (for strains overproducing LysLW32, LysJavan488 and LysPollywog) and grown under the same conditions until they reached an OD_600nm_ of 0.8 (for LysP7951) or 0.4 (for LysLW32, LysJavan488 and LysPollywog), after which they were subjected to a thermic shock (42°C) for 30 min in a shaking water bath. Cultures were then incubated for 3 h at 37°C (LysP7951) or overnight at 16°C (LysLW32, LysJavan488 and LysPollywog). Samples were collected and total extracts prepared for analysis by SDS-PAGE and Western blot with anti-His_6_-peroxidase antibody (Roche Applied Sciences). Total T_0_ and T_1_ extracts were prepared by recovering cells from culture samples immediately before and after protein production, followed by 10-fold concentration in Laemmli buffer and cell lysis by boiling. For analysis of protein solubility, culture samples were 40-fold concentrated in lysis buffer (50 mM HEPES, 500 mM NaCl, 50 mM imidazole, 1% glycerol, pH 7) supplemented with 20 μg/mL DNase, 10 mM MgCl_2_ and 1× Complete Mini EDTA-free Protease Inhibitor Cocktail (Roche Applied Sciences), and then lysed by sonication. Lysates were centrifuged, the supernatant recovered (S, soluble extract) and the pellet dissolved in a solution containing 50 mM Tris.Cl pH 8.8, 7 M urea and 2 M thiourea (I, insoluble extract).

### Endolysin purification.

Induction of protein production was performed as described in the previous section. Cells were then collected by centrifugation (8 000 *g*, 15 min, 4°C) and resuspended in 1/40 volumes of lysis buffer. Cell disruption was done by sonication (Vibra Cell, Sonic Materials) with 10 cycles of 15 s pulses (amplitude 50%) intercalated with 45 s pauses on an ice bath. The lysate was then centrifuged (30 000 *g*, 30 min, 4°C) and the His_6_-tagged proteins in the supernatant purified by metal chelate affinity chromatography (HisTrap HP column, GE Healthcare) as described previously (30). Selected peak fractions were then subjected to gel filtration chromatography using a HiLoad 16/60 Superdex 75 pg column (GE Healthcare) and protein buffer as mobile phase (same composition of the lysis buffer but without imidazole). Protein fractions were analyzed by SDS-PAGE and quantified by the Bradford method (Bio-Rad Protein Assay, Bio-Rad Laboratories) using bovine serum albumin or γ-globulin as standards. Proteins were aliquoted and stored at −80°C until use.

### Analytical gel filtration.

Samples of purified proteins were applied to a Superose 6 10/300 GL column (GE Healthcare) equilibrated in protein buffer and run at a flow rate of 0.4 mL/min. The column void (V_0_) and total (V_t_) volumes were determined using the elution volumes of 1 mg/mL blue dextran 2000 (GE Healthcare) and 4% acetone, respectively, prepared in protein buffer. The molecular masses and Stokes radii (Rs) of the proteins of interest were estimated as described before (31), using the partition coefficient as $K_{\mathrm{av}}=\frac{V_{e-V_{0}}}{V_{t-V_{0}}}$, where V_e_ is the elution volume of the protein. Protein standard (Bio-Rad Laboratories and GE Healthcare) included thyroglobulin (molecular mass of 670 kDa; Rs of 8.6 nm), γ-globulin (158 kDa; 5.1 nm), conalbumin (75 kDa; 3.72 nm), ovalbumin (44 kDa; 2.73 nm), myoglobin (17 kDa; 1.91 nm) and vitamin B_12_ (1.35 kDa; 0.85 nM). Reconstitution of LysP7951 multimer was done by 1 h incubation at 37°C of defined amounts of each individually purified subunit, immediately before gel filtration.

### Study of endolysin lytic activity.

Lytic activity of purified endolysins was tested against target bacterial species by spotting different enzyme quantities in dense lawns of viable cells. Bacteria were grown until an OD_600nm_ of approximately 0.8. S. pyogenes M6 (ST382) was grown at 37°C in brain heart infusion (BHI) in a 5% carbon dioxide atmosphere. S. thermophilus 4078 was grown at 42°C in M17 medium supplemented with 1.5% lactose under anaerobic conditions (anaerobic jar with AnaeroGen sachets, ThermoFisher Scientific). Cells were collected by centrifugation, recovered in 1/100 volumes of fresh growth medium (for S. pyogenes) or phosphate-buffered saline supplemented with 16% glycerol and stored at −80°C until use (for S. thermophilus). The cell suspension was then 100-fold diluted in reaction buffer supplemented with 0.7% agar (final concentration) and poured into a petri dish. LysJavan488 reaction buffer was as described elsewhere with slight modifications (32, 33) - 35 mM ammonium acetate, 150 mM NaCl and 10 mM CaCl_2_, pH 6.25. LysP7951 reaction buffer was optimized (Fig. S8) to 25 mM HEPES, 5 mM CaCl_2_, pH 7. 10-microliter drops of protein samples were spotted on the plates and, after overnight incubation under the conditions indicated above, they were observed for the presence of lysis halos.

Activity of LysP7951 was also evaluated in liquid medium. S. thermophilus 4078 was grown as above until an OD_600nm_ of approximately 0.4, collected by centrifugation, washed with phosphate-buffered saline and resuspended in reaction buffer to an OD_600nm_ of approximately 0.4. The ability of LysP7951 and its mutants to cause lysis of S. thermophilus was evaluated under static conditions at 37°C by making hourly OD_600nm_ measurements in a Varioskan LUX Multimode Microplate Reader (Thermo Fisher Scientific).

### Protein analyses by mass spectrometry (MS) and small-angle X-ray scattering (SAXS).

**(i) Denatured protein analyses**. Samples were buffer exchanged into water, using Bio-Rad Micro Bio-Spin 6 columns, by performing multiple cycles of buffer exchange and centrifugation. Proteins were diluted to approximately 5 μM in 1:1 water:acetonitrile solution with 1% formic acid prior to analysis. Proteins were analyzed by electrospray in positive mode on a Bruker FTICR 7 T SolariX XR mass spectrometer equipped with a Paracell. Spectra were acquired in magnitude mode with 1 MB size. For LysP7951 CTP, 25 transients were acquired at *m/z* 500 – 2,000 and, for LysP7951 FLP, 50 transients were acquired at *m/z* 1,000 – 2,400. The instrument was externally calibrated with Na/TFA clusters. Spectra analysis was performed with DataAnalysis 5.0 (Bruker) using the SNAP 2 algorithm to calculate the proteins monoisotopic masses.

**(ii) Native MS analysis.** The purified LysP7951 protein complex was buffer exchanged into 150 mM ammonium acetate, using Bio-Rad Micro Bio-Spin 6 columns, by performing multiple cycles of buffer exchange and centrifugation. It was then diluted to a final concentration of approximately 10 μM. Native mass spectrometry analysis was performed on a modified Micromass Q-TOF Ultima II (MSVision), using nano-electrospray Ionization (nano-ESI)-MS in positive mode. Samples (4 μL) were electrosprayed using gold-coated borosilicate emitter tips prepared in-house using a micropipette puller from Sutter Instrument (Sutter P1000). Ionization conditions were fine tuned for each sample, most notably capillary voltage and collision cell pressure. Typical parameters were 1.4 – 1.6 kV capillary, cone 137 V, source temperature 100°C, and collision cell pressure of 3.0 × 10^−2^ mbar. Collision energy for complex stability analysis was set to 0, 15 and 100 V. In total, around 500 spectra were accumulated per final spectrum. The mass spectrometer was externally calibrated with cesium iodide clusters (25 mg/mL). UniDec software (34) was used for data analysis.

**(iii) SAXS analysis**. We collected synchrotron SAXS data from LysP7951 CTP at the BM29 beamline at ESRF (Grenoble, France). For a concentration series of 1.00, 1.85, 2.70 mg/mL in protein buffer, scattering patterns were recorded in 1-s frames at a wavelength of 0.99 Å using a Pilatus 1M (DECTRIS) detector and a sample-detector distance of 2.812 m, covering a momentum transfer (*s*) range of 0.00378 to 0.51792 Å^−1^. All frames were measured at 20°C with no radiation damage or aggregation signs. Scattering patterns of the buffer were also recorded before and after each protein sample. Final curves at each concentration were derived after the averaged buffer scattering pattern was subtracted from the protein sample patterns. Further processing was performed using the ATSAS 3.0 software suite (35). The radius of gyration, *Rg*, was evaluated using Guinier's approximation. By indirect Fourier Transform, we obtained the *P(r)* distribution function that we then used to generate the low-resolution *ab initio* molecular envelope for LysP7951 CTP with DAMMIF of the package ATSAS 3.0. Briefly, 20 independent envelopes were generated, superimposed, and averaged to assess their robustness and define the most populated volume. We processed the averaged *ab initio* models with DAMFILT (36) to remove low occupancy and loosely connected parts. SAXS data and model are available at SASBDB (www.sasbdb.org) under the project “SAXS studies on phage endolysin LysP7951” with the accession code SASDNU5.
