# Supplementary material for: On the Occurrence and Multimerization of Two-Polypeptide Phage Endolysins Encoded in Single Genes
Source: Microbiol Spectr. 2022 Jul 25;10(4):e01037-22. doi: 10.1128/spectrum.01037-22 (PMC9430671; doi:10.1128/spectrum.01037-22)

Supplemental figures to:

On the occurrence and multimerization of two-polypeptide phage endolysins encoded in single genes

Daniela Pinto[1,*], Raquel Gonçalo[1], Mariana Louro[2], Marta Sousa Silva[2], Guillem Hernandez[3], Tiago N. Cordeiro[3], Carlos Cordeiro[2] and Carlos São-José[1,*]

[1] Research Institute for Medicines (iMed.ULisboa), Faculdade de Farmácia da Universidade de Lisboa, Av. Prof. Gama Pinto, 1649-003 Lisboa, Portugal

[2] Laboratório de FT-ICR e Espectrometria de Massa Estrutural, MARE – Marine and Environmental Sciences Centre, Faculdade de Ciências da Universidade de Lisboa, 1749-016 Lisboa, Portugal

[3] Instituto de Tecnologia Química e Biológica António Xavier, Universidade Nova de Lisboa, Av. da República, 2780-157 Oeiras Portugal

* To whom correspondence should be addressed. Tel: +351 217 946 420; Email: dspinto@farm-id.pt; csaojose@ff.ulisboa.pt

Pinto *et al*.
On the occurrence and multimerization of two-polypeptide phage endolysins encoded in single genes

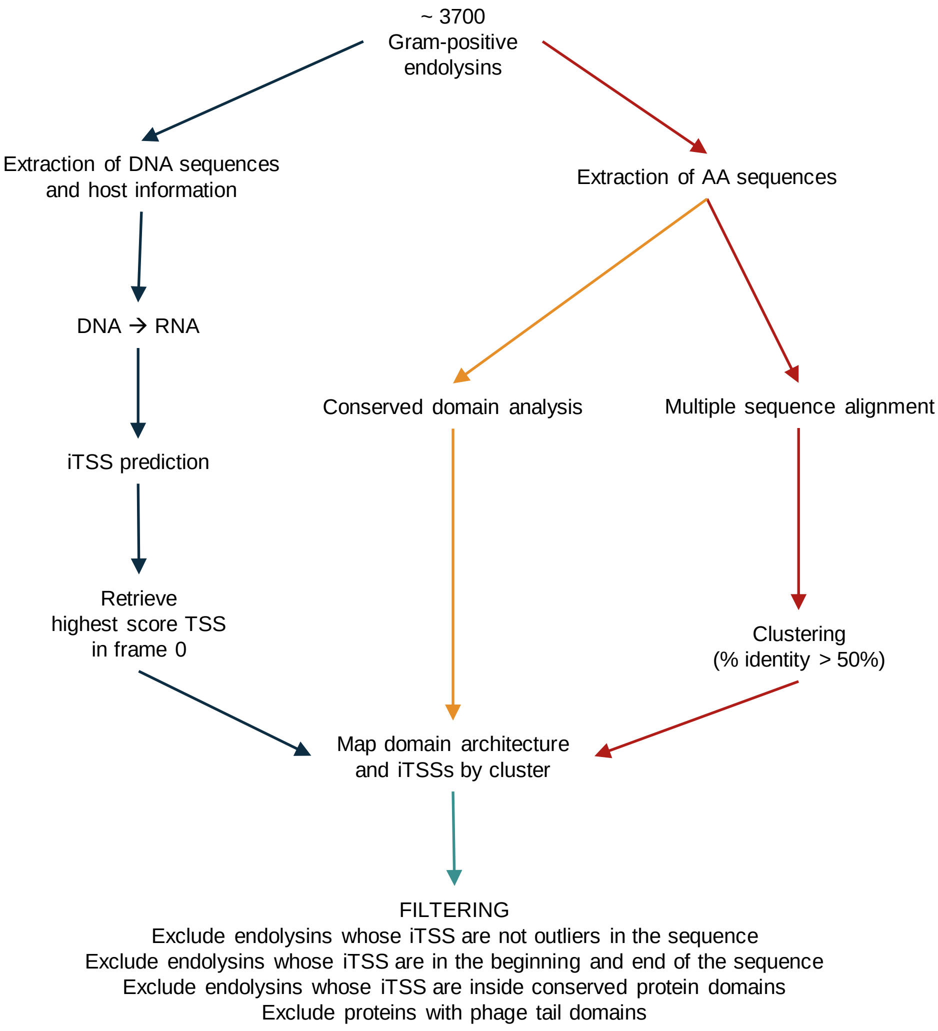

Figure S1. Bioinformatics workflow.

Schematic representation of the bioinformatics workflow applied to identify endolysin genes with two in frame overlapping ORFs. Additional details can be found in the methods section.

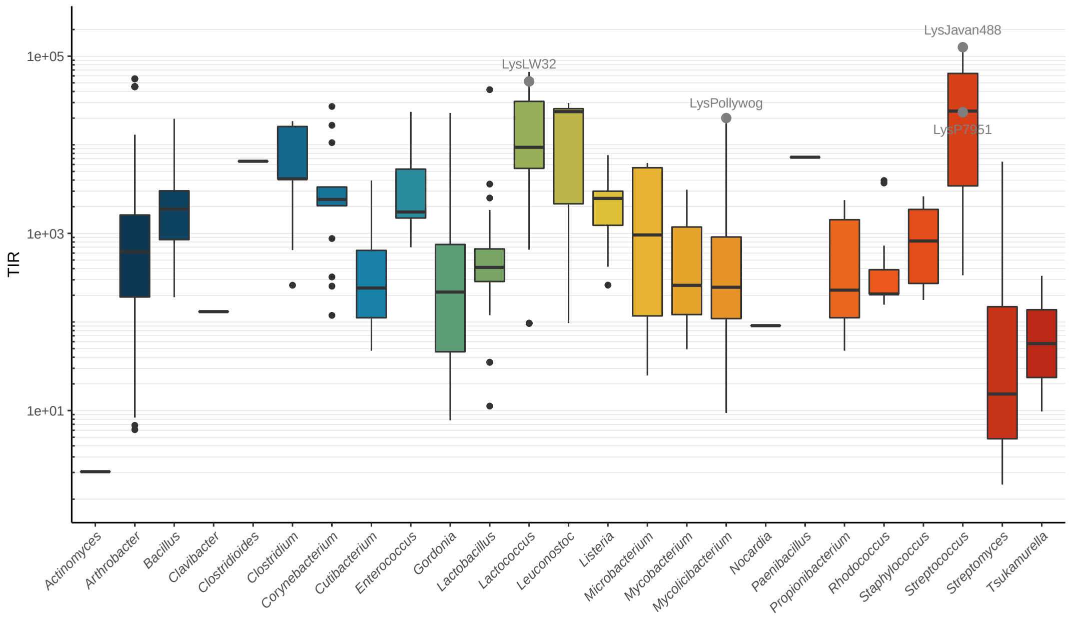

Figure S2. Predicted TIR (translation initiation rates) of the endolysins selected.

The boxplots represent the distribution of the maximal predicted TIR (translation initiation rate) for the putative in frame iTSSs (internal translation start sites) found in endolysin genes of phages infecting the indicated genera. The TIRs of the selected endolysins for experimental analysis are indicated by the large, grey closed circles for comparison.

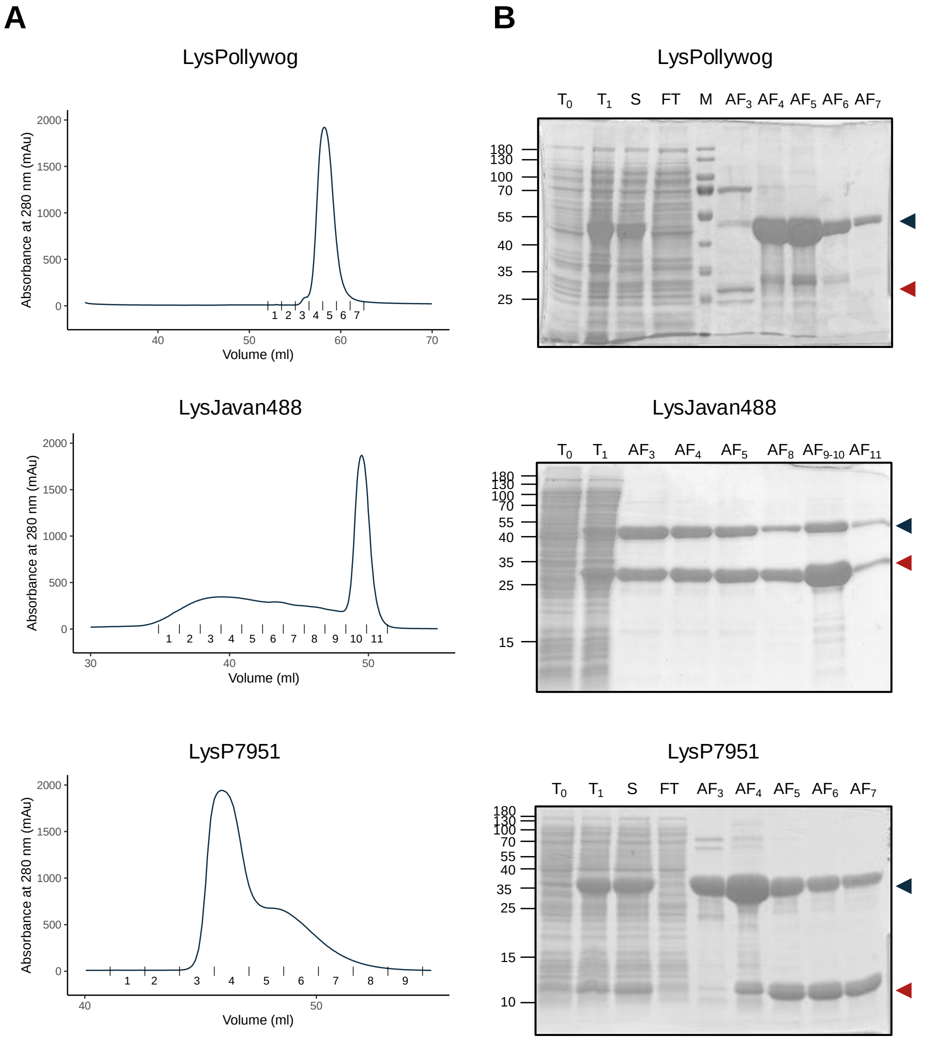

Figure S3. Endolysin purification by metal chelate affinity chromatography (AF).

A. Protein elution profile from the AF column. The numbers shown on the bottom correspond to the fractions collected for further analysis. **B.** SDS-PAGE analysis of the AF fractions. The numbers shown on the side of each gel image refer to the molecular masses of the protein marker in kDa. T_0, total cell extract before protein production induction; T_1, total cell extract after protein production; S, soluble fraction (*i.e.*, extract subjected to AF); FT, AF flow through (*i.e.*, unbound proteins); AF_n, AF fraction number n as indicated on the corresponding elution profile (**A**). Blue and red arrowheads indicate the FLP and CTP polypeptides, respectively.

Pinto *et al*.
On the occurrence and multimerization of two-polypeptide phage endolysins encoded in single genes

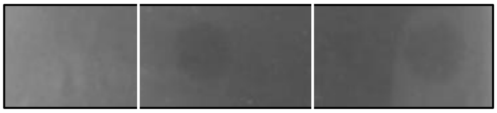

Figure S4. Lytic activity of the two isoforms of LysJavan488.

Lytic activity of the LysJavan488 FLP and CTP was evaluated by spotting 16 µM of protein on a dense lawn of S. pyogenes cells.

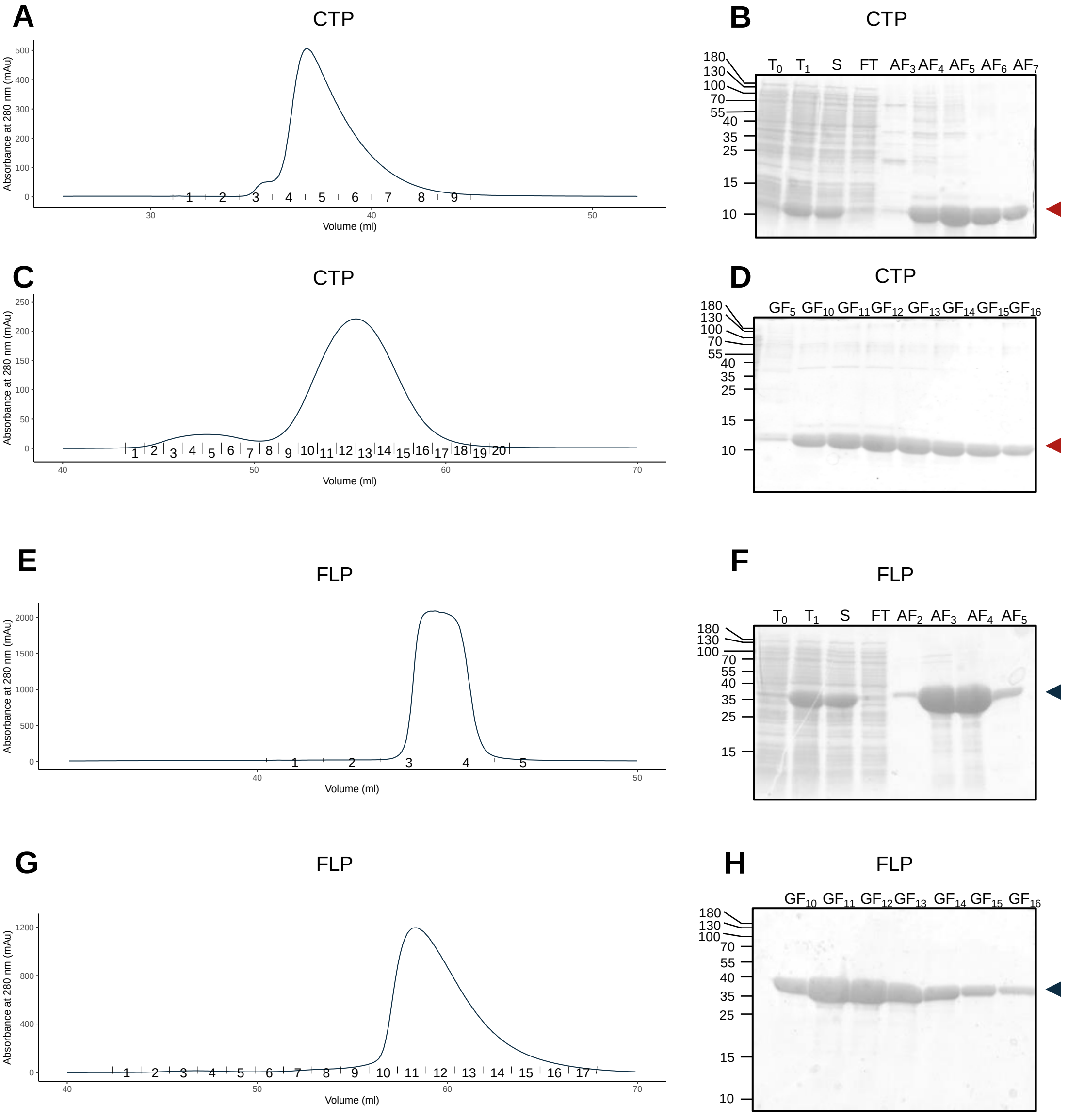

Figure S5. Purification of the independently produced LysP7951 FLP and CTP polypeptides.

Protein elution profiles of the AF chromatography are shown in **A** and **E**. Elution profiles of the fractions AF_4-AF_6 of $LysP7951_{209\text{-}310}$ and AF_3-AF_4 of $LysP7951_{M209L}$ subjected to GF chromatography are shown in **C** and **G**. The numbers shown on the bottom of the chromatograms correspond to the fractions collected for further analysis. SDS-PAGE analysis of the collected fractions are shown in **B**, **D**, **F** and **H**. The numbers shown on the side of each gel image refer to the molecular masses of the protein marker in kDa. T_0, total cell extract before protein production induction; T_1, total cell extract after protein production; S, soluble fraction (*i.e.*, extract subjected to AF); FT, AF flow through (*i.e.*, unbound proteins); AF_n, AF fraction number n as indicated on the corresponding elution profile; GF_n, GF fraction number n as indicated on the corresponding elution profile. Blue and red arrowheads indicate the FLP and CTP polypeptides, respectively

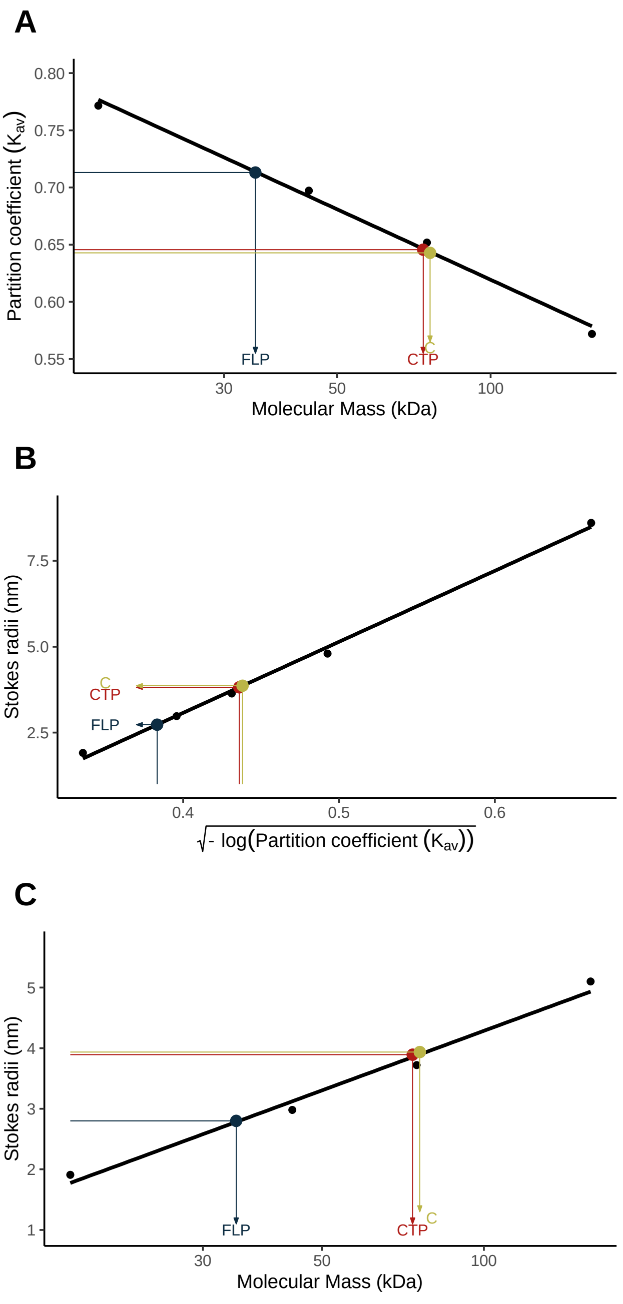

Figure S6. Molecular mass estimation from the analytical GF data.

A. Correlation between the partition coefficient (Kav) and the molecular mass (kDa) of the proteins of the standard. **B.** Correlation between the Stokes radii (nm) and the square root of the negative logarithm of the partition coefficient of the proteins of the standard. **C.** Correlation between the stokes radii (nm) and the molecular masses (kDa) of the proteins of the standard. The large blue, red and green dots represent the position of LysP7951$_{M209L}$ (FLP), LysP7951$_{209\text{-}310}$ (CTP) and LysP7951 (Complex), respectively, on each regression line.

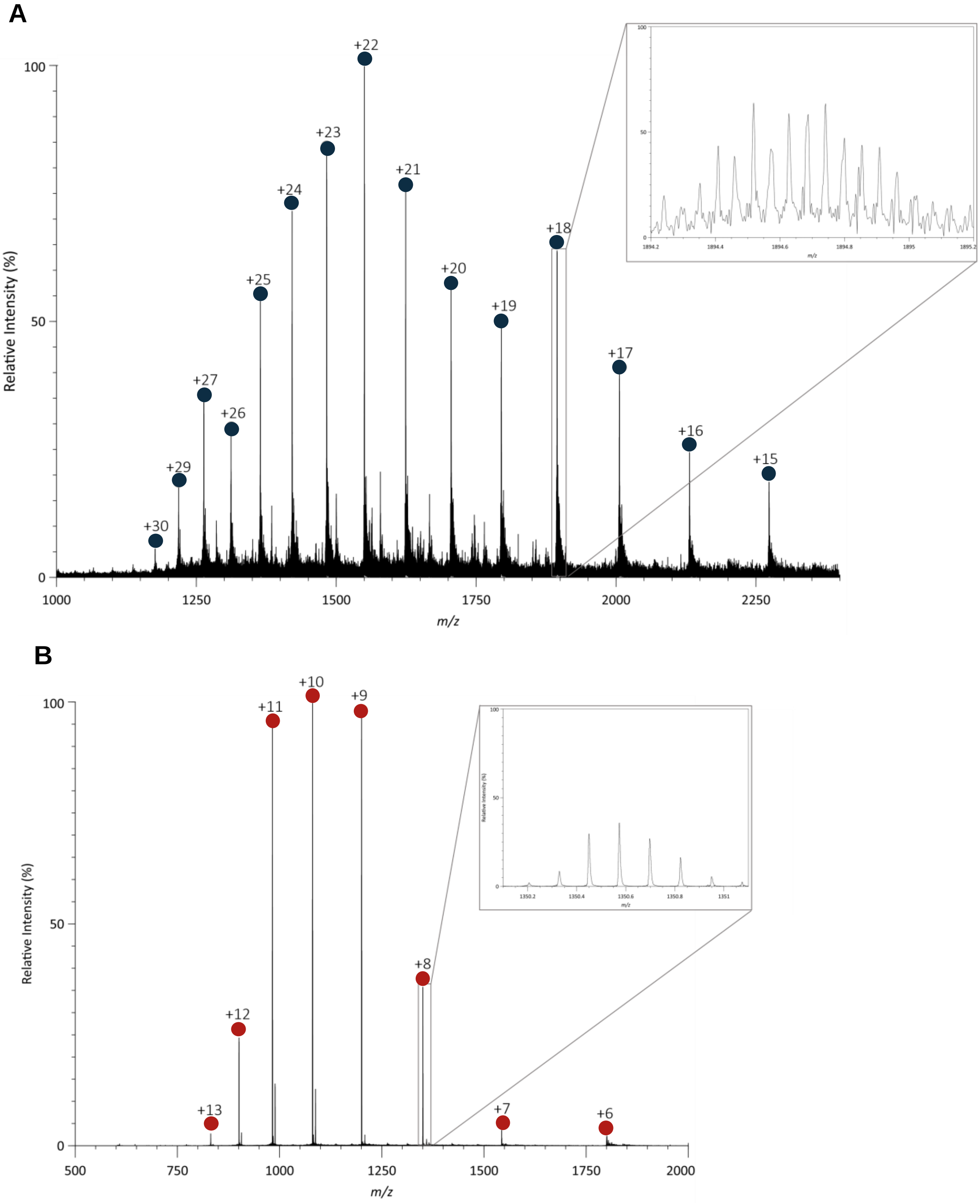

Figure S7. Denatured mass spectrometry analysis of LysP7951 protein species.

A. Spectrum acquired in denaturing conditions of the FLP. This protein generated a CSD at m/z 1,100 – 2,300 with +30 to +15 charges and an experimental mass of 34,063.70 Da. **B.** Spectrum acquired in denaturing conditions of the CTP, with a CSD at m/z 800 – 1,800 (+13 to +6 charges) and 10,790.40 Da.

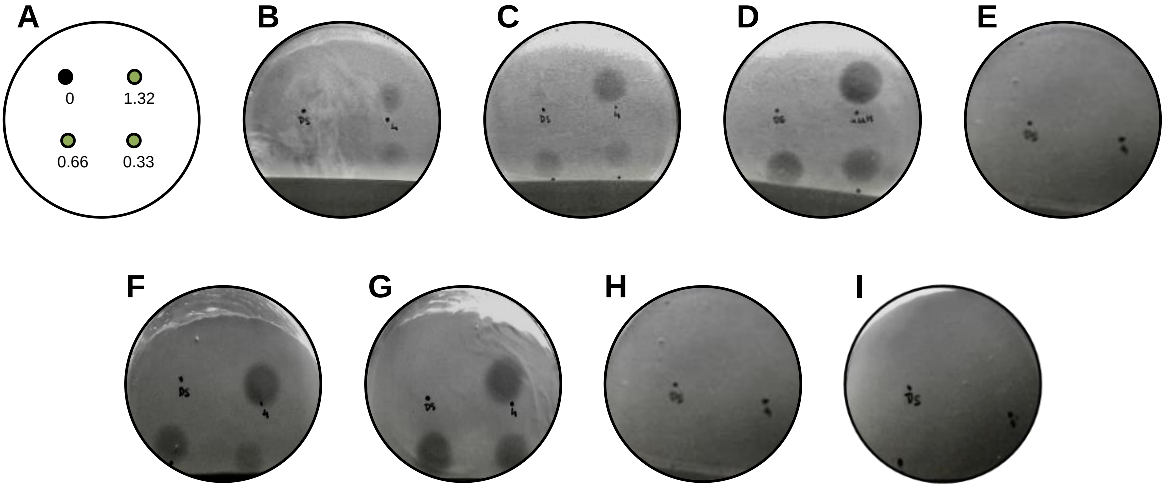

Figure S8. Activity of LysP7951 against *S. thermophilus* in different buffers.

A. Schematic representation of the distribution of the LysP7951 samples in the plates. The numbers indicate the concentration in μM. “0” corresponds solely to the proteins buffer. **B.** Activity in 25 mM Hepes, 5 mM $CaCl_2$, pH 8. **C.** Activity in 25m M Hepes, 5 mM $CaCl_2$, 250 nM NaCl, pH 7. **D.** Activity in 25 mM Hepes, 5 mM $CaCl_2$, pH 7. **E.** Activity in 25 mM Hepes, 5 mM $CaCl_2$, 500 mM NaCl, pH 7. **F.** Activity in 25 mM Hepes, 5 mM $CaCl_2$, 500 mM NaCl, 1 mM TCEP, pH 7. **G.** Activity in 25mM Pipes, 5 mM $CaCl_2$, pH 6. **H.** Activity in 50 mM Sodium acetate/acetic acid buffer, 5 mM $CaCl_2$, pH 5. **I.** Activity in 50 mM Sodium acetate/acetic acid buffer, 5 mM $CaCl_2$, pH 4. Transparent halos indicate lysis of *S. pyogenes* by LysP7951.

Figure S9 (see next pages). Schematic representation of endolysins with annotated domain architectures and mapping of in frame iTSSs.

Domain architecture (ordered by average maximal TIR of cluster)

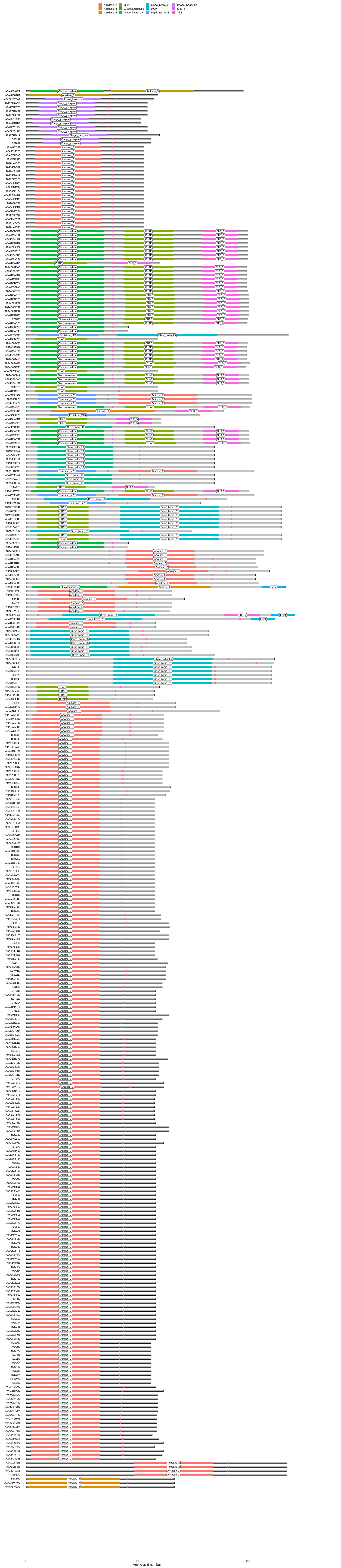

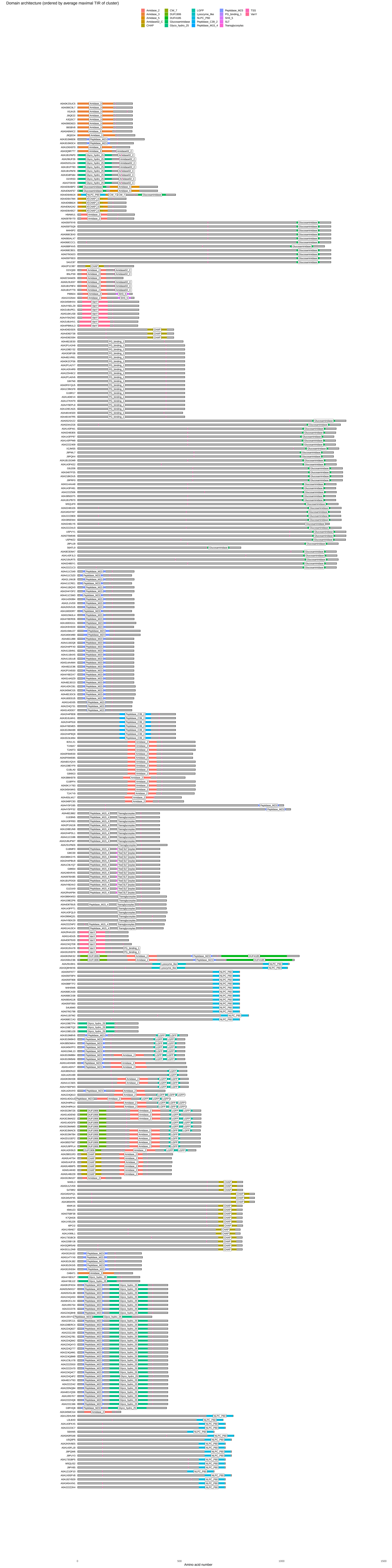

Domain architecture (ordered by average maximal TIR of cluster)
Amidase_2
Amidase_3
Amidase_5
Amidase02_C
CHAP
CW_7
DUF1906
DUF4185
Glucosaminidase
Glyco_hydro_25
LGFP
Lysozyme_like
NLPC_P60
Peptidase_C39_2
Peptidase_M15_4
Peptidase_M23
PG_binding_1
SH3_5
SLT
Transglycosylas
TSS
VanY
0
500
1000
1500
Amino acid number

Domain architecture (ordered by average maximal TIR of cluster)

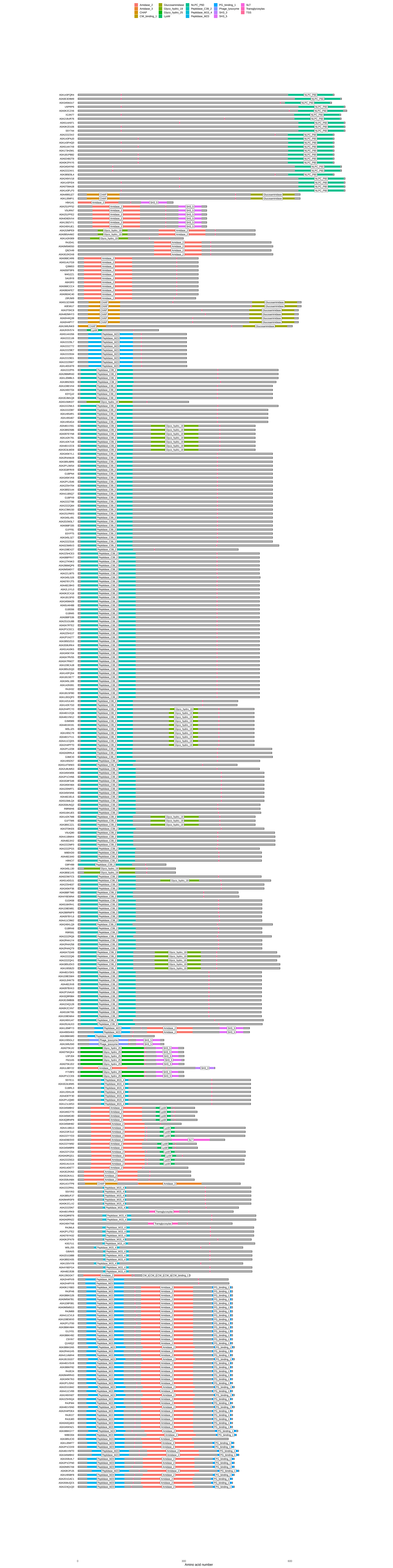

Domain architecture (ordered by average maximal TIR of cluster)

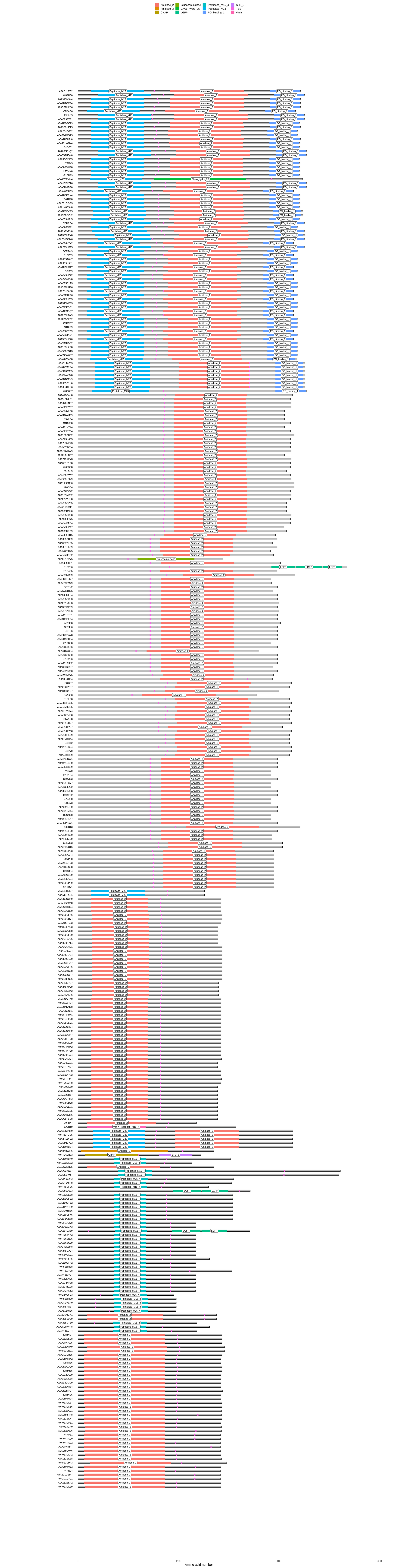

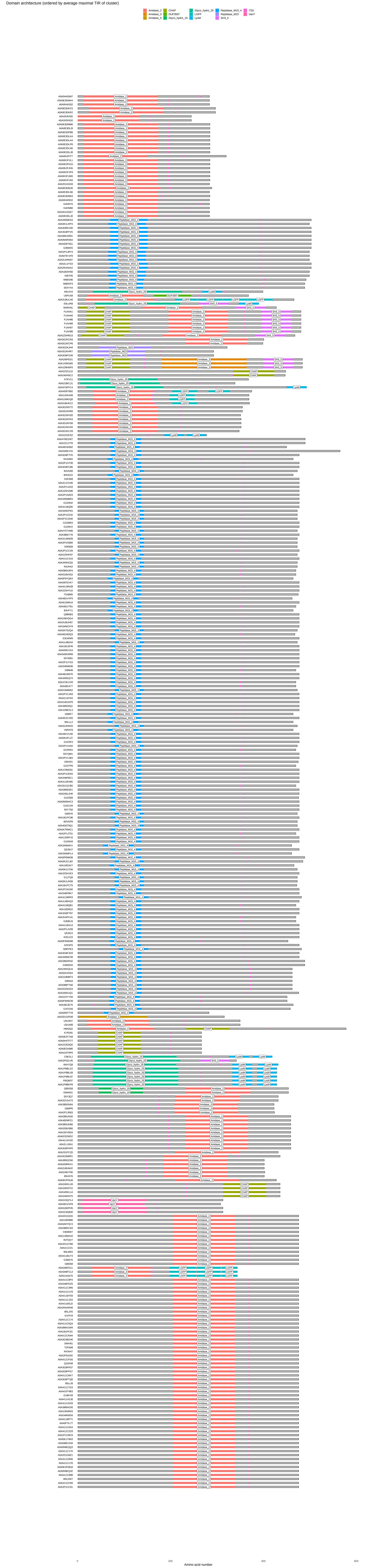
Domain architecture (ordered by average maximal TIR of cluster)
Amidase_2
Amidase_3
Amidase_5
CHAP
DUF3597
Glyco_hydro_19
Glyco_hydro_25
LGFP
LysM
Peptidase_M15_4
Peptidase_M23
SH3_5
TSS
VanY
0
200
400
600
Amino acid number

Domain architecture (ordered by average maximal TIR of cluster)

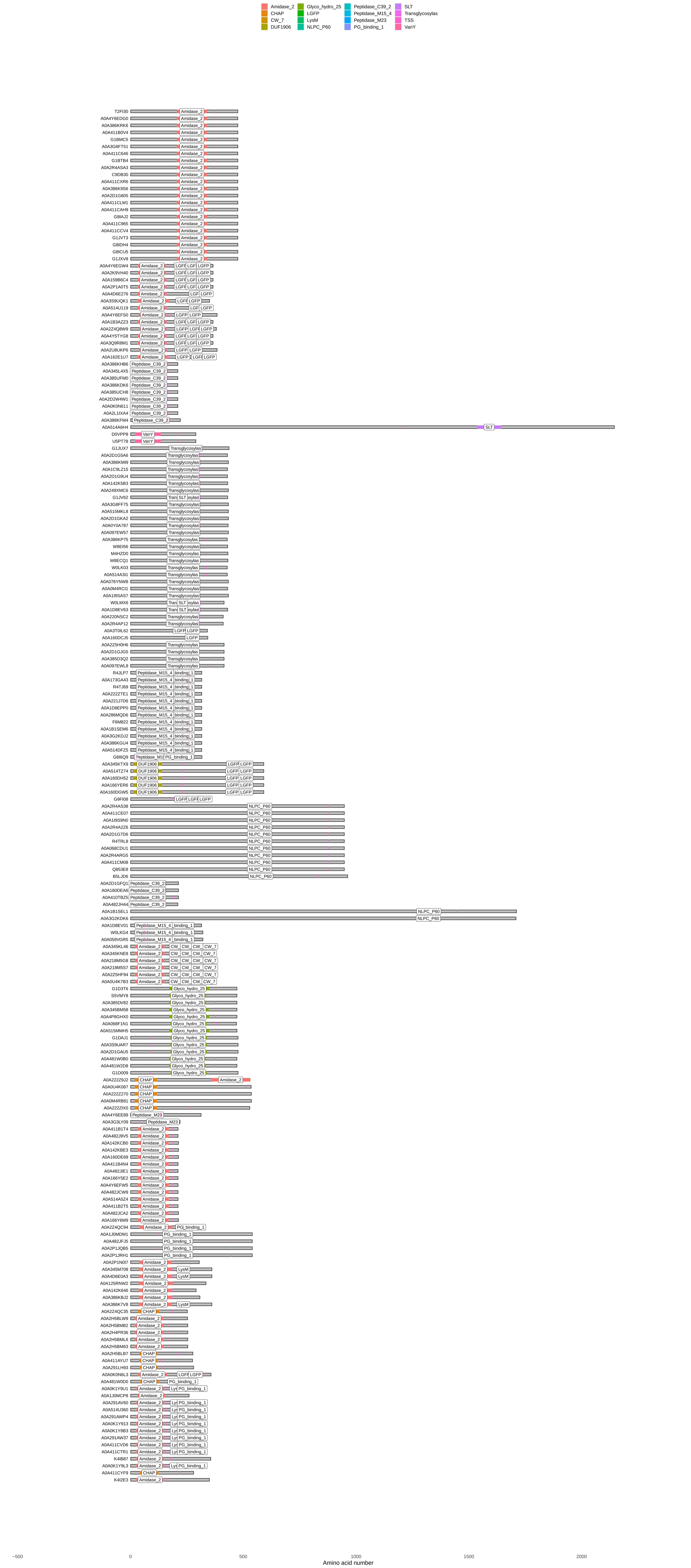

Supplement: Supplemental file 1 — Supplemental material. Download spectrum.01037-22-s0001.pdf, PDF file, 1.1 MB [file spectrum.01037-22-s0001.pdf]
